# Deep homology of a *brachyury* cis-regulatory syntax and the evolutionary origin of the notochord

**DOI:** 10.1126/sciadv.adw3307

**Published:** 2025-07-25

**Authors:** Tzu-Pei Fan, Jun-Ru Lee, Che-Yi Lin, Yi-Chih Chen, Ann E. Cutting, R. Andrew Cameron, Jr-Kai Yu, Yi-Hsien Su

**Affiliations:** ^1^ Institute of Cellular and Organismic Biology, Academia Sinica, Taipei, Taiwan.; ^2^ Division of Biology and Biological Engineering, California Institute of Technology, Pasadena, CA, USA.; ^3^ Marine Research Station, Institute of Cellular and Organismic Biology, Academia Sinica, Yilan, Taiwan.

## Abstract

Expression of *brachyury* in the notochord is regarded as a chordate novelty and links to the origin of the notochord, yet the evolution of this regulatory control remains unclear. Here, we uncovered a regulatory syntax (named SFZE) consisting of binding sites for four transcription factors in notochord enhancers of chordate *brachyury* genes. SFZE was also identified in potential *brachyury* enhancers in various non-chordate animals and even in *Capsaspora*, a unicellular relative to animals. These non-chordate SFZE-containing enhancers exhibited activity in the zebrafish notochord. Furthermore, the SFZE syntax in a non-chordate confers endoderm activity. Our results indicate the ancient association of SFZE with *brachyury*, likely predating the origin of animals. The emergence of notochordal *brachyury* expression could be attributed to co-option of upstream signals acting on the conserved SFZE syntax, which facilitates the origin of the notochord from rudimentary endodermal cells.

## Teaser

An ancient gene regulatory code facilitates the origin of the notochord from rudimentary gut cells by recruiting upstream factors.

## INTRODUCTION

The notochord, a defining feature of chordates (amphioxus, tunicates, and vertebrates), plays critical roles in chordate development. The notochord cells differentiate from the axial mesoderm, which arises from the organizer located dorsal to the blastopore at gastrulation onset. During gastrulation, the notochord progenitors signal to surrounding tissues, shaping the basic chordate body plan (*1*). The two closest relatives of chordates are the hemichordates and echinoderms (together ambulacrarians); these three groups constitute the deuterostomes. The hemichordate stomochord, an anterior rod-like protrusion from the pharynx, was once considered homologous to the chordate notochord. However, molecular studies do not support stomochord-notochord homology (*2*). In a protostome annelid, it was shown that a population of midline mesodermal cells expresses a set of notochord-specific genes and differentiates into a medial ventral longitudinal muscle named the axochord (*3*). While the axochord has been proposed to be a notochord homolog, it remains possible that the two structures may have evolved convergently (*4*).

*Brachyury* encodes a T-box transcription factor (TF) essential for notochord development (*5*). It may represent the most ancient T-box family member, with orthologs found in various animals and non-animals (*6*). Non-chordate *brachyury* genes can transform ascidian endodermal cells into notochord cells (*7*), indicating protein functional equivalence between chordates and non-chordates. In contrast to the conservation of protein function, *brachyury* expression patterns differ between chordates and non-chordates: In chordates, the expression pattern is generally conserved. For example, *brachyury* transcripts in amphioxus and frog are first detected in cells surrounding the blastopore. Subsequently, the *brachyury*-expressing cells internalize during gastrulation to form the notochord (*8*, *9*). In non-chordate bilaterians, a circumblastoporal expression domain is also observed in gastrulae; *brachyury* is additionally expressed in the hindgut and oral ectoderm, but not in axial mesodermal cells (*10*). These observations have led to a hypothesis that the circumblastoporal expression of *brachyury* is an ancestral trait in bilaterians, while axial mesodermal expression represents a chordate-specific innovation. Therefore, the evolutionary origin of the notochord likely associates with the gain of a novel *brachyury* expression domain in the axial mesoderm (*11*). Emergence of new expression domains is often attributed to births or modifications of enhancers [or cis-regulatory modules (CRMs)]. Studies in ascidian and zebrafish have identified CRMs upstream of the *brachyury* translation start site (TSS) that drive reporter gene expression in the notochord (*12*–*14*). It remains to be determined whether these notochord enhancers evolved de novo in the chordate ancestor or have a more ancient origin.

## RESULTS

### Ambulacrarian *brachyury* BACs are active in zebrafish

**Fig. 1.**
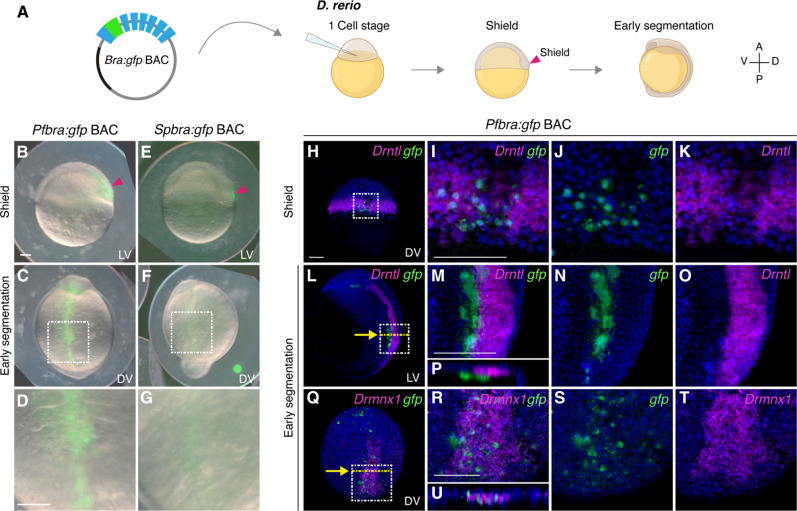
*Pfbra:gfp* BAC activates GFP expression in the organizer and the hypochord of zebrafish embryos. (**A**) Circular *Pfbra*/*Spbra*:*gfp* BACs were each introduced into zebrafish zygotes, and GFP signals were observed at the shield and early segmentation stages (A, anterior; P, posterior; V, ventral; D, dorsal). (**B** to **G**) GFP signals in zebrafish embryos injected with *Pfbra*:*gfp* BAC or *Spbra*:*gfp* BAC. GFP signal in the dorsal margin (shield) at the shield stage is indicated by the red arrowhead. The white dashed boxes in (C) and (F) are magnified and shown in (D) and (G), respectively. (**H** to **U**) Double fluorescent in situ hybridization of *gfp* (green) and *Drntl* [magenta in (H) to (P)] or *Drmnx1* (magenta in Q to U) in *Pfbra:gfp* BAC-injected zebrafish embryos at indicated stages. Nuclei were counterstained with Hoechst 33342 (blue). The areas in the white dashed boxes in (H), (L), and (Q) are enlarged and shown in (I), (M), and (R); single-channel images are shown in (J) and (K), (N) and (O), and (S) and (T), respectively. [(P) and (U)] The *XZ* sections along the yellow dashed lines are indicated with yellow arrows in (L) and (Q). The orientation of embryos is indicated in each panel: LV, lateral view; DV, dorsal view. All scale bars represent 100 μm. Panels that are in the same scale: (B), (C), (E), and (F); (D) and (G); (H), (L), and (Q); (I) to (K), (M) to (P), and (R) to (U). Created in BioRender. Fan, T. (2025) https://BioRender.com/n8z3595.

To explore the evolutionary origin of the *brachyury* notochord enhancer, we analyzed CRMs of *brachyury* genes from animals that are closely related to chordates, including the hemichordate *Ptychodera flava* and the sea urchin *Strongylocentrotus purpuratus*. The *gfp* knock-in bacterial artificial chromosomes (BACs) harboring the *brachyury* ortholog of either *P. flava* or *S. purpuratus* (*Pfbra*/*Spbra*:*gfp* BACs) recapitulated endogenous *brachyury* expression in their cognate embryos, driving expression in the blastopore, oral ectoderm, and endoderm (figs. S1 and S2) (*15*, *16*), although some ectopic expression was also observed. These results suggest that the BACs contain CRMs sufficient to drive *brachyury* expression at the analyzed stages. To examine CRM activities of the ambulacrarian *brachyury* genes in chordates, we introduced each of the reporter BACs into zebrafish zygotes. At the onset of gastrulation (shield stage), 6.5 hours post fertilization (hpf), ~33% of the zebrafish embryos injected with the *Pfbra*:*gfp* BAC showed green fluorescent protein (GFP) signals in the embryonic shield, which is equivalent to the dorsal organizer that gives rise to midline structures, including the notochord (*17*). By the early segmentation stage (11 to 14 hpf), ~45% of the transgenic embryos showed GFP signal in the dorsal midline (Fig. 1, A to D, and table S2). In contrast to the strong reporter activity driven by *Pfbra*:*gfp* BAC, *Spbra*:*gfp* BAC produced weak or undetectable GFP signals, with only two of 174 embryos showing GFP expression in the organizer at the shield stage, and the signals became faint at the early segmentation stage (Fig. 1, E to G, and table S2). The lower activity of the sea urchin BAC could be due to the presence of repressive elements, lower compatibility of the sea urchin promoter in zebrafish embryos, and potential modifications of regulatory sequences (experiments shown later). Double fluorescent in situ hybridization confirmed that *gfp* expression driven by *Pfbra*:*gfp* BAC localized to *Drntl* (*18*) (no tail, zebrafish *brachyury* ortholog)–expressing cells in the shield (Fig. 1, H to K), while, at the early segmentation stage, *gfp* transcripts were detected in the dorsal midline underneath the *Drntl*-expressing notochord cells (Fig. 1, L to P). The GFP signal was confirmed to be in the hypochord [marked by *Drmnx1* expression; (*19*)], a midline structure sharing the same progenitors with the notochord (Fig. 1, Q to U, and fig. S3) (*17*). Together, these results indicate that the ambulacrarian *brachyury* CRMs are responsive to the notochord progenitor regulatory state but not to those of the notochord at later segmentation stage.

### Hemichordate PfCRM2 is active in the notochord

**Fig. 2.**
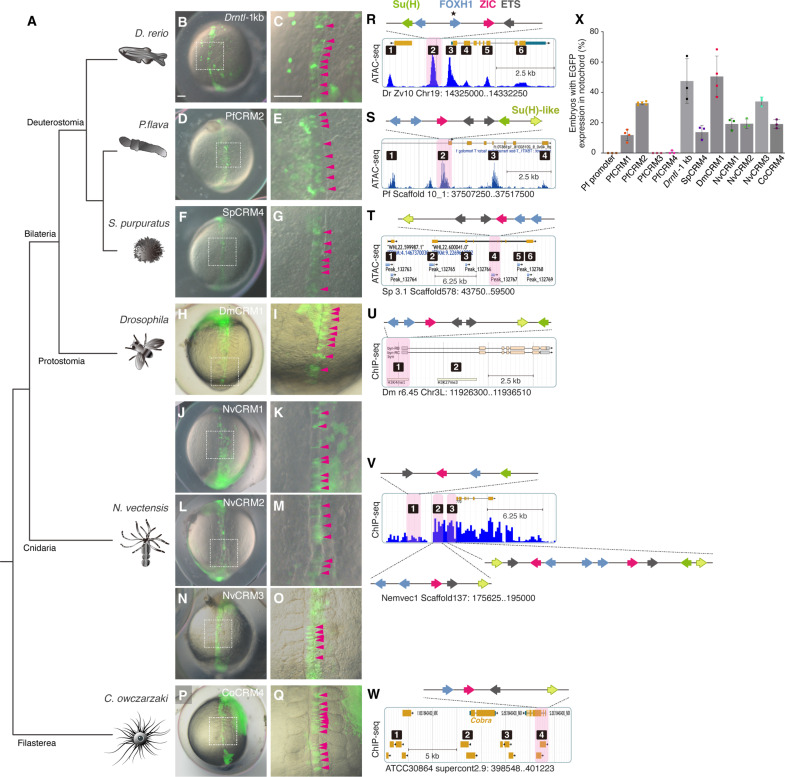
*Brachyury* CRMs with SFZE/SFZE-like syntax show activity in the zebrafish notochord. (**A**) Phylogenetic relationships of the analyzed species. (**B** to **Q**) Zebrafish embryos injected with SFZE/SFZE-like containing CRMs from zebrafish (*Drntl*-1kb), hemichordate (PfCRM2), sea urchin (SpCRM4), fruit fly (DmCRM1), sea anemone (NvCRM1, NvCRM2, and NvCRM3), and filasterea (CoCRM4). The right panels show magnified views of the notochord region (white dashed boxes in the corresponding left panels) with magenta arrowheads indicating enhanced GFP (EGFP) signals in the notochord cells. The left panels are in the same scale, as are the right panels. Scale bars in (B) and (C), 100 μm. (**R** to **W**) The putative CRMs based on published ATAC-seq (Drntl, Pfbra, and Spbra loci) and chromatin immunoprecipitation sequencing (ChIP-seq) datasets (Dmbyn, H3K4me1, and H3K27me3; Nvbra, H3K4me1; Cobra, H3K4me1, and H3K4me3). Potential CRMs are indicated with numbers, and CRMs carrying SFZE/SFZE-like motifs are shaded in pink. The TF binding sites are denoted schematically with colored arrows placed on top of each diagram to show the orientation and order. Colors of TF sites are shown in (R) and (S). Genome versions, scaffold/chromosome numbers, and positions of the genomic loci displayed in (R) to (W) are indicated below the respective panel. The samples used for generating of the ATAC-seq and ChIP-seq datasets are listed in table S7. (**X**) Percentages of embryos with EGFP signals in the notochord out of EGFP-positive embryos. Each colored square represents the result of one experiment. Gray columns represent the average of at least three biological replicates, with error bars serving as SDs.

To identify CRM(s) of *Pfbra* contributing to the transcriptional activities in zebrafish embryos, we analyzed ATAC-seq (assay for transposase accessible chromatin using sequencing) data to locate open chromatin regions at the *Pfbra* locus. Among the four major ATAC-seq peaks, PfCRM2 spans 717–base pair (bp) immediately upstream of the TSS and contains the promoter (Fig. 2S). We constructed reporters for PfCRM2 and the other three PfCRMs fused with the deduced *Pfbra* promoter (a 332-bp fragment upstream of the TSS), using an *egfp* vector (fig. S4A) (*20*). For comparison, constructs containing *Pfbra* promoter alone or the known notochord enhancer of *Drntl* [~1 kb upstream of the *Drntl* TSS (*Drntl*-1kb)] (*12*) were also generated (fig. S4A). At the shield stage, *Drntl*-1kb and PfCRM2 drove *egfp* expression in the hypoblast of the embryonic shield (notochord precursor) in most embryos (98% for *Drntl*-1kb, 80% for PfCRM2). Enhanced GFP (EGFP) signals were also observed in the dorsal germ ring for some embryos (fig. S4, B, C, H, and I; and table S3). Reporters with the *Pfbra* promoter alone or with PfCRM1, PfCRM3, or PfCRM4 exhibited lower activities in the hypoblast (fig. S4, D to N, and table S3). At the early segmentation stage, *Drntl*-1kb and PfCRM2 drove *egfp* expression in the notochord in 49 and 33% of EGFP-positive embryos, respectively (Fig. 2, B to E and X). In contrast, *Pfbra* promoter alone or with the other CRMs showed minimal or no activity in the notochord (Fig. 2X; fig. S4, O to V; and table S3). The EGFP-positive cells in the PfCRM2-injected embryos were *Drntl*-expressing notochord cells (fig. S4, W to Z). These results reveal that, among the four PfCRMs, only PfCRM2 exhibits comparable transcriptional activities in the hypoblast and notochord to those of *Drntl*-1kb. Thus, it appears that PfCRM2 shares similar regulatory features with the zebrafish notochord enhancer. Additionally, the *Pfbra* promoter has limited regulatory function in the hypoblast, likely accounting for the hypoblast activities of PfCRM1 and PfCRM4. Furthermore, the observation that PfCRM2, but not *Pfbra*:*gfp* BAC, is transcriptionally active in the notochord implies the presence of silencer(s) within the BAC that suppress PfCRM2 activity in the notochord. PfCRM3 likely serves as a silencer, given that it significantly reduced *Pfbra* promoter activity in the hypoblast and the absence of its activity in the notochord.

### SFZE syntax of *brachyury* notochord enhancers

To identify the regulatory features underlying the transcriptional activity in the notochord, we compared sequences of PfCRM2 with the known *brachyury* notochord enhancers from zebrafish (*12*) and the ascidian *Ciona intestinalis* type A (or renamed as *Ciona robusta*) (*21*, *22*). Using mVISTA (*23*), we were unable to detect conserved regions between PfCRM2 and the notochord enhancers (fig. S5). Nevertheless, the ability of PfCRM2 to drive reporter expression in the notochord suggests that TF binding sites within PfCRM2 are sufficient to respond to particular TFs present in the zebrafish notochord. Studies on the regulatory controls of zebrafish and *Ciona brachyury* genes have identified TF binding sites critical for expression in the notochord. These include a Foxh1 site located within *Drntl*-1kb (*12*) and two sites for Suppressor of Hairless [Su(H)] within the proximal enhancer of the *Ciona brachyury* (*Cibra*) (*13*); one complies the consensus sequence (GTGRGAR) (*24*), while the other is less ideal (NTGRGAR). Additionally, a shadow enhancer located ~800 bp upstream of the *Cibra* TSS contains two Ets and one ZicL sites in a face-to-face orientation essential for enhancer activity in the notochord (*21*). Similarly, in another ascidian, *Halocynthia roretzi*, Ets and ZicN in the promoter region of *brachyury* (*Hrbra*) are required for *Hrbra* initiation in notochord precursors (*14*). We thus reasoned that rather than sequence conservation, TF sites with specific grammar may be essential for transcriptional activity in the notochord. *Drntl*-1kb encompasses two ATAC-seq peaks (DrCRM2 and DrCRM3 in Fig. 2R). Within DrCRM2, we found binding sites for Zic, Ets, and Su(H) adjacent to the previously identified Foxh1 site (Fig. 2R and fig. S6) (*12*); an additional Foxh1 site was also observed. The Zic and Ets sites of DrCRM2 are facing each other (Fig. 2R and fig. S6), akin to the shadow enhancer of *Cibra* (*21*). Additionally, we found two Foxh1 sites adjacent to the previously identified ZicL site in the *Cibra* shadow enhancer (figs. S6 and S7). In sum, both notochord enhancers share a specific syntax: Binding sites of the four TFs are arranged in a defined order [5′-Su(H)-Foxh1-Foxh1-Zic-Ets-3′ for zebrafish and reversed in *Ciona*] and with specific orientations (face-to-face orientation of Zic and Ets sites, and the same direction for Zic and the adjacent Foxh1 site). These TF sites are also in close proximity to each other, especially the Foxh1-Zic-Ets sites (span less than one nucleosome length, ~147 bp; table S4). This tight spacing likely facilitates collaborative TF recruitment for regulating *brachyury* expression. Hereafter, we refer to this arrangement as the “SFZE” syntax, based on the initials of the four TFs. This syntax likely represents a functional unit for driving *brachyury* expression in the notochord. In line with this hypothesis, we were unable to identify the syntax in the *Xenopus brachyury* (*Xbra*) promoter, which activates reporter in the blastopore but not in the notochord (*25*). Together, our results suggest that the SFZE syntax is conserved in notochord enhancers of chordate *brachyury* genes.

### Deep homology of the SFZE syntax

To explore whether the SFZE syntax has an ancient origin and might account for the notochord activity of the hemichordate PfCRM2, we scanned PfCRM sequences for binding sites of the four TFs. We discovered that PfCRM2 contains two Foxh1, one Zic, two Ets, and two Su(H) sites [one is a less optimal Su(H)-like site], organized similarly to the chordate SFZE syntax (Fig. 2S and fig. S6). One difference is that the Su(H) sites in PfCRM2 are adjacent to Ets sites but to Foxh1 sites in zebrafish SFZE syntax. Thus, the notochord activity of PfCRM2 is likely attributed to the presence of the syntax, with FZE serving as the core, and the position of Su(H) sites is less critical. PfCRM3 does not contain Ets and Su(H) sites, and PfCRM4 lacks Su(H) sites, consistent with the absence of notochord activity. PfCRM1 contains all four TF sites, but the Ets and Zic sites are in the same orientation. Consistent with this slightly disrupted syntax, PfCRM1 exhibited lower notochord activity than PfCRM2 but higher activity than PfCRM3 and PfCRM4 (Fig. 2X and fig. S6). We further examined the sequences of the sea urchin *Spbra* CRMs. Among the putative CRMs, SpCRM4 (located in the third intron) harbors a SFZE motif. It complies with the FZE orientation rule but is partially inverted compared to the SFZE syntax (i.e., SEZF; Fig. 2T and fig. S6). Nevertheless, SpCRM4 exhibited transcriptional activity in the zebrafish notochord, albeit at a lower level (Fig. 2, F, G, and X; and table S5). These results strongly suggest the role of the syntax in the notochord activity and demonstrate that the association of SFZE with *brachyury* is present in non-chordate deuterostomes, predating the origins of the notochord.

To further trace the origin of the SFZE syntax, we analyzed potential CRMs of *brachyury* orthologs of *Drosophila melanogaster* (*Dmbyn*), a protostome, and sea anemone *Nematostella vectensis* (*Nvbra*), a non-bilaterian. We identified SFZE in DmCRM1 that mirrors the organization in hemichordate PfCRM2 (Fig. 2U and fig. S6). Furthermore, DmCRM1 drove *egfp* expression in the zebrafish notochord, with activity comparable to that of zebrafish reporter (Fig. 2, H, I, and X; and table S5). For *Nematostella*, we found that NvCRM1 contains an inverted FZE motif upstream of a Su(H) site, while these sites within NvCRM2 violate the FZE orientation rules. NvCRM3 has two consecutive SFZE motifs. The upstream motif has an inverted FZE syntax that matches the motif order within sea urchin SpCRM4. Meanwhile, the downstream motif deviates from the FZE grammar rules, with Ets and Zic sites oriented in the same direction. Additionally, these sites in NvCRM1, NvCRM2, and the downstream SFZE motif of NvCRM3 span more than one nucleosome length (table S4). Correspondingly, NvCRM3 exhibited higher transcriptional activity in zebrafish notochord than NvCRM1 and NvCRM2 (Fig. 2, J to O and X, and table S5). Its activity exceeded that of SpCRM4, likely due to a synergistic effect of the two SFZE motifs.

The deeply conserved SFZE syntax within animals led us to extend this analysis to the *brachyury* ortholog of the filasterean *Capsaspora owczarzaki* (*Cobra*) (*26*), a unicellular eukaryote that is phylogenetically close to metazoans. Within one potential CRM (CoCRM4), we found an SFZE syntax containing one Su(H)-like site with the FZE core located within an exon of a gene (CAOG_05510) neighboring *Cobra* (CAOG_05512) (Fig. 2W and fig. S6). This nonanimal CoCRM4 also drove *egfp* expression in zebrafish notochord (Fig. 2X and table S5). Together, these results showed the association of the SFZE syntax with *brachyury* orthologs of a wide range of animals and a unicellular eukaryote. Furthermore, CRMs with SFZE syntax exhibited transcriptional activities in zebrafish notochord. Our results thus strongly support the conclusion that the SFZE syntax is deeply conserved and it likely originated before the emergence of animals.

### Foxh1 and Ets sites are functionally important

**Fig. 3.**
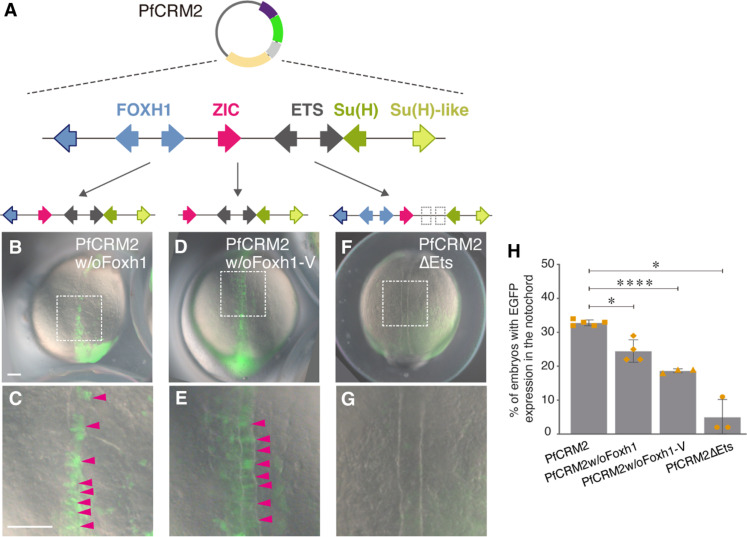
Foxh1 and Ets sites of the hemichordate SFZE syntax are functionally important for notochord activity. (**A**) Diagram of the reporter constructs of PfCRM2 with deletions of the Foxh1 sites or mutations of the Ets sites. The additional Foxh1 site in the vector is indicated by the leftmost blue arrow with black outline. (**B** to **G**) Representative images show zebrafish embryos injected with the truncated or mutated PfCRM2 reporter constructs. Magnifications of the notochord regions highlighted by white boxes in (B), (D), and (F) are shown in (C), (E), and (G), respectively. EGFP signals in the notochord cells are indicated with magenta arrowheads. Embryos are oriented in the dorsal view with anterior to the top. Top panels [(B), (D), and (F)] and bottom panels [(C), (E), and (G)] are in the same scale as indicated in (B) and (C), respectively. w/o, without; delta symbol (△), mutation. All scale bars represent 100 μm. (**H**) Percentages of zebrafish embryos exhibiting EGFP signals in the notochord among EGFP-positive embryos. Each yellow data point represents the result of a single experiment. The gray columns are average results from at least three biological replicates, with error bars showing SDs. *P < 0.05; ****P < 0.0001.

Nodal and fibroblast growth factor (FGF) signals activate zebrafish *brachyury* expression in the notochord via Foxh1 and Ets TFs, respectively (*12*, *27*, *28*). To evaluate the importance of Foxh1 and Ets sites within the hemichordate SFZE syntax, we removed these sites in PfCRM2. Deletion of the Foxh1 sites significantly reduced reporter activity (Fig. 3, A to E and H), and mutation of the Ets sites strongly decreased reporter activity in the notochord (Fig. 3, F to H). These results indicate that both Foxh1 and Ets sites in PfCRM2 are functional and likely respond to endogenous Nodal and FGF signaling in the notochord. Additionally, the Ets sites play a crucial role, while the Foxh1 sites confer an additive effect, strengthening the transcriptional activity in the notochord.

### Sea urchin SFZE syntax confers endoderm activity

**Fig. 4.**
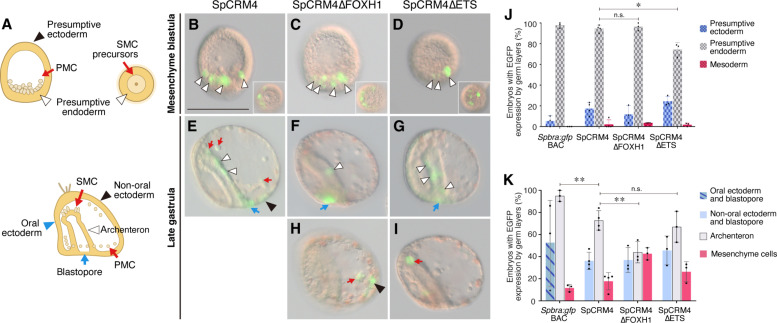
Foxh1 and Ets sites of the sea urchin SFZE syntax confer endodermal activity at different developmental stages. (**A**) Illustrations of sea urchin embryos at the mesenchyme blastula (top left, lateral view; top right, vegetal view) and late gastrula (bottom) stages. Colored arrows and arrowheads mark different embryonic territories. (**B** to **I**) Representative EGFP-positive embryos showing reporter activities. Colored arrows and arrowheads indicate EGFP-positive cells within the territories shown in (A). Embryos are oriented as in (A). For late gastrulae, the ventral side is to the left. Insets in (B) to (D) are the vegetal view to show EGFP signals in the presumptive endoderm. delta symbol (△), mutation. Panels are all in the same scale [scale bar in (B) 100 μm]. (**J** and **K**) Percentages of mesenchyme blastula (J) and late gastrula (K) embryos showing EGFP signals in the indicated territories. PMC, primary mesenchyme cells; SMC, secondary mesenchyme cells. *P < 0.05; **P < 0.01; n.s., not significant.

To this point, we had shown that non-chordate *brachyury* CRMs with SFZE have notochord activity in zebrafish embryos; however, their endogenous roles remained unknown. To explore the function of the SFZE syntax in organisms lacking a notochord, we analyzed SpCRM4 activity during sea urchin gastrulation (Fig. 4A). The promoter driven by SpCRM4 exhibited strong activity in the presumptive endoderm of mesenchyme blastula (i.e., initiation of gastrulation) (Fig. 4, B and J), recapitulating the endogenous *Spbra* expression (fig. S8, A to D), while *Spbra* promoter alone showed negligible background activity (table S6). By late gastrula stage, the SpCRM4 reporter was active in the archenteron, non-oral ectoderm, cells surrounding the blastopore, and mesenchymal cells (Fig. 4, E and K). The ectodermal expression differed from the endogenous pattern, suggesting that additional CRMs are required. Mutation of the two Foxh1 sites had no effect at the mesenchyme blastula stage but significantly decreased reporter signals in the archenteron. In contrast, disruption of the two Ets sites decreased the activity in the presumptive endoderm significantly but had little effect at late gastrula stage (Fig. 4, C to D and F to K). These results indicate that SpCRM4 functions mainly as an endoderm enhancer at the onset of gastrulation and behaves as a general enhancer across three germ layers during gastrulation. Moreover, the endodermal activity of SpCRM4 at the onset and end of gastrulation partially depends on the respective Ets and Foxh1 sites within the syntax. Given the similarity of endodermal expression patterns of *brachyury* in sea urchin, sea star, and hemichordate (fig. S8), the endodermal activity of the SFZE syntax is likely conserved in ambulacrarians.

### SFZE syntax in vertebrate *brachyury* paralogs

*Brachyury* is generally a single copy gene in invertebrates (*29*). After two rounds of whole-genome duplications (2R) during vertebrate evolution (*30*) following lineage-specific gene loss, ray-finned fishes have *tbxta* and *tbxtb* genes (*31*), while tetrapods only retain *tbxtb* (*29*, *31*). Because the association of SFZE with *brachyury* predates 2R, we anticipate that both vertebrate paralogs would have inherited SFZE. In zebrafish, the identified syntax is associated with *tbxta* (*Drntl*). At the zebrafish *tbxtb* locus (*Drbra*), we uncovered the syntax in two putative CRMs situated 4.8 kb upstream and 12 kb downstream of the TSS (figs. S6 and S7). In mouse *brachyury* (*Mmbra*/*T*/*tbxtb*), a SFZE-containing CRM (1.6 kb upstream of the TSS) lies within a 8.3-kb region that directs *Mmbra* expression in the primitive streak (homologous to the blastopore) but not in the notochord of the mouse embryo (*32*). In *Xenopus*, two putative CRMs containing SFZE syntax were identified. One is 140.8 kb upstream and the other 13 kb downstream of the *Xbra*/*tbxtb* TSS (figs. S6 and S7); activities of these two CRMs remain unknown. These findings demonstrate that the SFZE syntax is present in both vertebrate *brachyury* paralogs, reinforcing the idea of its ancestral association with *brachyury* and retention after 2R.

## DISCUSSION

In this study, we show that the regulatory features of functionally equivalent enhancers can be preserved by specific motif grammar. We uncover a conserved SFZE syntax associated with *brachyury* of species across a wide range of taxa, including chordates and non-chordates, and even a nonanimal unicellular eukaryote. The proximity spacing, order, and orientation of the TF sites are maintained despite adjacent sequence divergence, suggesting the deeply conserved regulatory code under strong evolutionary constraints.

**Fig. 5.**
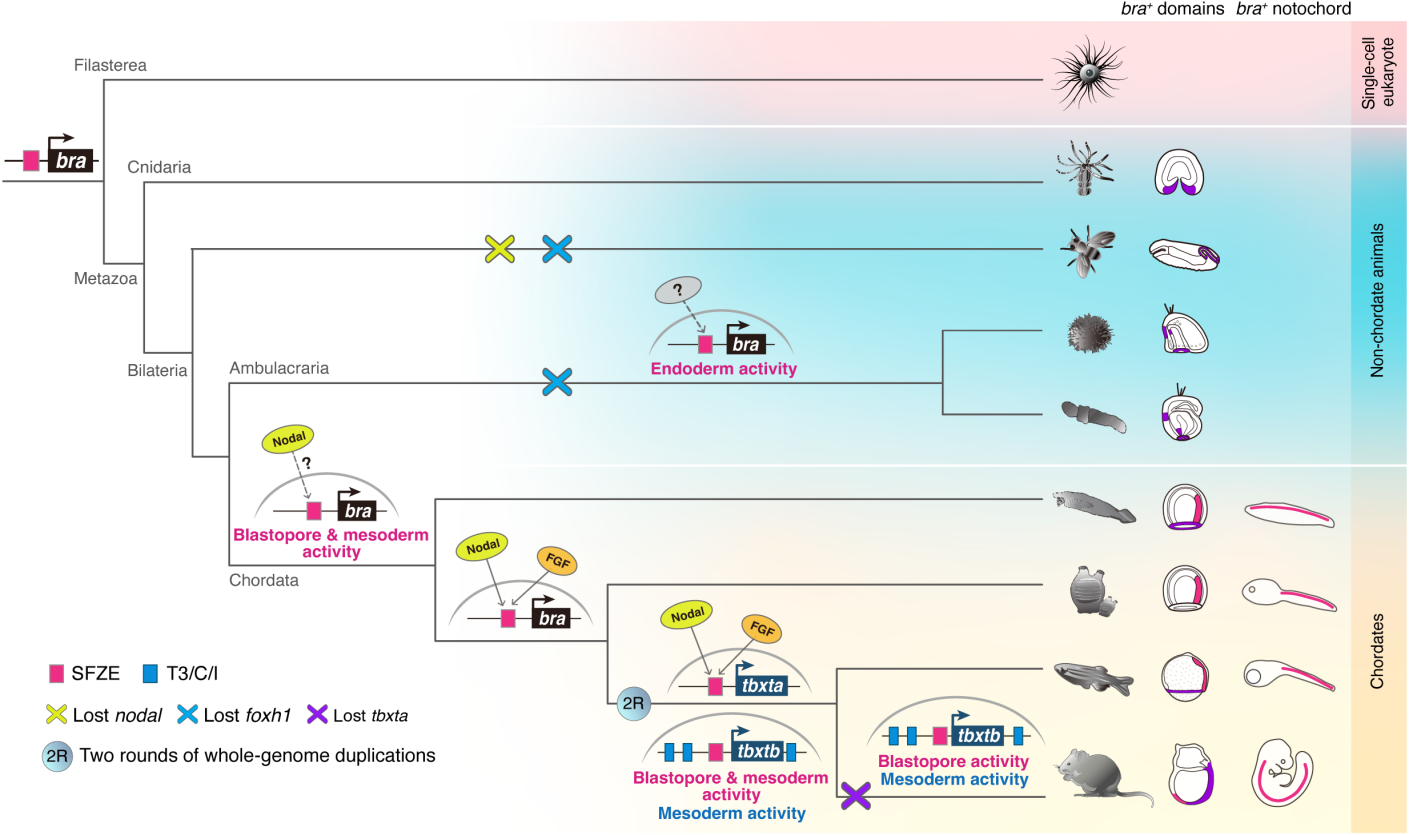
Proposed scenario of SFZE evolution and the origin of the notochord. The SFZE syntax (red rectangles) associated with *brachyury* orthologs could have already been present before the emergence of animals. The regulatory network that activates *brachyury* in the axial mesoderm by concurrent Nodal and FGF signals through the SFZE syntax may have been established in the chordate lineage, at least before the divergence of tunicates and vertebrates. The Ets and Fox family factors (activated by yet to be determined signaling pathways, indicated by question marks) likely regulate *brachyury* expression through SFZE in at least ambulacrarians. Some components of the regulatory inputs could have been modified due to the loss of *nodal* and/or *foxh* genes in *Drosophila* and ambulacrarians (cross symbols). After two rounds of whole-genome duplications (2R), the SFZE syntax remained associated with both *brachyury* paralogs. Other notochord enhancers (T3/C/I) were subsequently evolved to control one of the paralogs (i.e., *tbxb*). In tetrapods, only *tbxb* was retained, and the notochord activity of the mammalian SFZE may have been substituted by T3/C/I enhancers. Created in BioRender. Fan, T. (2025) https://BioRender.com/14o3zg1.

At the onset of gastrulation, *brachyury* expression in ambulacrarians and chordates preferentially localized on the ventral and dorsal blastoporal margins, respectively, which aligns with the hypothesis of the dorsoventral inversion in the chordate ancestor (*33*, *34*). Our results showed that the SFZE-containing CRMs have transcriptional activity in the zebrafish dorsal margin (part of the future blastopore) and sea urchin blastopore, suggesting a conserved regulatory role of the syntax in the blastoporal region of deuterostomes. During gastrulation, chordate *brachyury* genes maintain expression in the dorsal axial region of the invaginating archenteron (presumptive notochord), whereas, in ambulacrarian, expression is restricted in a few ventral endodermal cells (fig. S8). The function of Nodal and FGF signals in controlling *brachyury* expression in the notochord appears to have arisen at least before the divergence of tunicates and vertebrates (Fig. 5). In the basal chordate amphioxus, the notochord expression of *brachyury* is controlled by Nodal (*35*, *36*), but it is not strongly affected when FGF signaling is inhibited (*37*). Nevertheless, within the potential notochord enhancers (*38*) of the two amphioxus *brachyury* paralogs (*Bfbra1* and *Bfbra2*), we found an SFZE motif in *Bfbra1* and two pairs of Su(H) binding sites in *Bfbra2* (fig. S6).

Unlike the synergistic effect of the Foxh1 and Ets sites in the zebrafish notochord, the endodermal activity of the two TF sites during sea urchin gastrulation is temporally decoupled. Intriguingly, *foxh* orthologs are absent in the sea urchin and hemichordate genomes (*39*, *40*). Additionally, inhibition of Nodal or FGF signals does not affect endodermal expression of sea urchin and hemichordate *brachyury* (*41*–*43*). Similarly, *nodal* and *foxh1* orthologs are missing in the *Drosophila* genome (*44*, *45*). It is likely that the Foxh1 sites of ambulacrarian and *Drosophila* are occupied by other Fox TFs (*46*, *47*), and the deeply conserved SFZE syntax may respond to different upstream factors. We thus propose that the notochord could have evolved from rudimentary endodermal cells, and, during chordate evolution, co-option of Nodal and FGF signals, via respective FoxH and Ets factors, reinforced *brachyury* expression in the dorsal axial region of the archenteron. Additional factors, such as Zic, orthologs of which are not expressed in the endodermal cells of sea urchin and hemichordate gastrulae (*48*, *49*), could also be recruited during chordate evolution. Further recruitment of notochord differentiation genes downstream of *brachyury* (*50*) would have also been necessary for notochord evolution.

Previous studies revealed that the 5′ promoter regions of *Cibra* and *Drntl*/*tbxta* are sufficient for *brachyury* expression in the notochord. However, the corresponding regions of *tbxtb* in mouse (*32*) and *Xenopus* (*25*) lack notochord activity, instead driving expression in the primitive streak/blastopore. Several notochord-specific enhancers (T3, C, and I) have been identified in the *tbxtb* loci of various jaw vertebrates (*51*, *52*). These conserved CRMs contain functional Brachyury sites for auto-regulation. However, T3, C, and I enhancers are absent in *tbxta* of ray-finned fishes, jawless vertebrates, and *Ciona*, suggesting an origin in the jawed vertebrate common ancestor (*52*). Notably, the notochord activity of T3, C and I is observed during somitogenesis in zebrafish, after the initial expression of *Drbra*/*tbxtb* in the dorsal margin prior to gastrulation (*31*). The early *Drbra*/*tbxtb* expression is thus likely regulated by the SFZE-containing CRMs that we identified. Intriguingly, the proximal enhancer of mouse *Mmbra* contains the SFZE syntax but is active only in the primitive streak. Thus, although SFZE-containing CRMs are active in both the blastoporal region and notochord during zebrafish gastrulation, their notochord activity may have been fully substituted by T3, C, and I enhancers in mammals.

Together, our study highlights the importance of conserved regulatory syntax in notochord evolution. The SFZE syntax has very deep evolutionary roots, likely originated before the emergence of animals. We propose that the ancestral SFZE syntax functions in endodermal cells during gastrulation. Subsequent co-option of upstream factors and functional divergence of the SFZE syntax after duplication shaped its activity during chordate evolution.

## MATERIALS AND METHODS

### Animal collection and embryo culture

Adult sea urchin *S. purpuratus* were obtained from Amro Hamdoun (University of California, San Diego) and kept at 15°C. Mature sea star *Archaster typicus* and hemichordate *P. flava* were collected during the breeding season (June to August and September to December, respectively) from Chito Bay, Penghu Islands, Taiwan. Spawning and embryo cultures were conducted as previously described (*53*–*55*) with modifications for *A. typicus*. In brief, starfish were weighed and injected with 1 ml of 200 μM 1-methyladenine per 100 g of weight on the dorsal side of arms to induce spawning. Starfish embryos were cultured at 33°C.

### Annotation of *brachyury* CRMs, promoters of *Pfbra* and *Spbra*, and the SFZE syntax

JBrowse (*56*) (version 1.16.10) was used to visualize the presumed CRMs at the *brachyury* loci of zebrafish, *P. flava*, *N. vectensis*, and *C. owczarzaki*. The processed datasets of ATAC-seq of zebrafish and the histone chromatin immunoprecipitation sequencing (ChIP-seq) of *N. vectensis* and *C. owczarzaki* were obtained from NCBI Gene Expression Omnibus under respective accession numbers GSE130944 (*57*), GSE46488 (*58*), and GSE71131 (*59*). The ATAC-seq dataset of hemichordate *P. flava* was obtained from a previous study and analyzed using standard pipelines (*60*). The sequencing reads were aligned to the cognate genome assemblies of zebrafish (Zv10) (*61*), *N. vectensis* (Nemvec1) (*62*), *C. owczarzaki* (C_owczarzaki_V2, accession number in the European Nucleotide Archive: GCA_000151315.2, www.ebi.ac.uk/ena/browser/view/GCA_000151315.2), and *P. flava* (ptychodera_flava version 1.0.114) (*63*) using Bowtie2 (version 2.5.1) (*64*). The ChIP-seq or the ATAC-seq datasets of mouse, *Xenopus*, *Ciona*, sea urchin, and *Drosophila* were from the NCBI Genome Data Viewer (*65*), Xenbase (v10.1) (*66*), ANISEED (*67*–*69*), Echinobase (*70*), and FlyBase (*71*), respectively. The promoters of *Pfbra* and *Spbra* were deduced by identifying positions of the core promoter elements (TATA box, initiator, downstream TFIIB recognition element, and downstream promoter element) (*72*, *73*) around the transcription start site, as well as the promoter-proximal elements (CCAAT and GC box) upstream of the core promoter elements (*74*). The SFZE motifs were identified by scanning the DNA sequences of putative CRMs to locate the binding sites of Zic and Foxh1, using the vertebrate PFMs (position frequency matrices) from the JASPAR database (*75*). The Ets and Su(H) binding sites were detected according to the consensus core sequences MGGAW (*76*, *77*) and GTGTRGAR (*24*), respectively. The suboptimal Su(H) binding site [Su(H)-like] was recognized as NTGRGAR or GTGRGAN.

### Generation of GFP knock-in BAC clones and EGFP reporter constructs

The *Spbra*:*gfp* BAC (BAC clone ID: Sp_117A03_L in the Echinobase) (*70*, *78*) contains an insert of ~131 kb of DNA, with *Spbra* located 24 kb from the end. The genomic DNA library of *P. flava* was constructed in the pBACe3.6 vector in Eric Davidson’s laboratory at California Institute of Technology. To identify BAC clones containing *Pfbra*, BAC arrayed library filters were screened with a digoxigenin (DIG)-labeled *Pfbra* probe (PCR DIG probe synthesis kit, Roche) following the hybridization protocol (DIG high prime DNA labeling and detection starter kit II, Roche). The DNA probe spans from 3′ of exon 6 to the flanking intron with a size of 173 bp. The identified *Pfbra* BAC clone was obtained from the Echinobase (clone position: plate #57, well J10) (*70*). The insert size of the *Pfbra* BAC was determined to be ~146 kb by digestion with Not I–HF (New England Biolabs) and separation via pulse-field gel electrophoresis. End sequencing and mapping to the *P. flava* genome revealed that *Pfbra* is located ~23 kb from the terminal end of the insert. To create a GFP knock-in BAC construct (*Pfbra*:*gfp* BAC), the *gfp* coding sequence was inserted at the TSS of *Pfbra* using homologous recombination (*79*, *80*). NucleoBond Xtra BAC plasmid purification kit was used to extract the BACs. Putative CRMs and promoters of *Pfbra* and *Spbra* were polymerase chain reaction (PCR) amplified from the BACs. CRMs of zebrafish, fruit fly, and *N. vectensis* were PCR amplified from the cognate genomic DNA. CoCRM4 was synthesized by BIOTOOLS Co. Ltd. The CRMs and promoters were constructed into the HLC (Hugo’s lamprey construct) reporter vector (*20*, *81*) by either restriction enzyme-mediated method, Gibson assembly (New England Biolabs), or combined by fusion PCR before ligating to the reporter vector. To modify particular TF binding sites in the SFZE syntax, the Q5 Site-Directed Mutagenesis Kit (E0554, New England Biolabs) was used to mutate the Foxh1 sites or the two guanine/cytosine nucleotides in the consensus core sequence (MGGAW) of the Ets sites. However, for the PfCRM2w/oFoxh1 construct, the 5′ end of the PfCRM2 was truncated by 224 bp to eliminate the two Foxh1 binding sites. The primer sets used in this study are listed in table S8.

### Microinjection

For microinjection of *P. flava*, zygotes were first pipetted for 10 s in filtered seawater (FSW) containing 0.25% *N*-acetyl-l-cysteine (NAC; Sigma-Aldrich) and incubated for 2 min to dissolve the jelly coat. The zygotes were then washed three times with FSW to remove NAC before being aligned on a protamine sulfate-coated culture dish for injection. The injection mixture used for *P. flava* included linearized *Pfbra*:*gfp* BAC (5 ng/μl), Dextran Alexa Fluor 555 (0.75 μg/μl; Invitrogen), and 0.12 M KCl. Microinjection of *S. purpuratus* and *Danio rerio* was conducted according to published procedures (*82*, *83*). For sea urchins, 9 pl of injection solution containing either linearized *Spbra*:*gfp* BAC (5 ng/μl) or PCR amplicons (0.4 ng/μl) of reporter constructs, Dextran Alexa Fluor 555 (0.75 μg/μl), and 0.12 M KCl was injected into the zygotes. Additionally, carrier DNA (15.4 ng/μl; Hind III digested *S. purpuratus* genomic DNA) was added to the solution to facilitate the incorporation of PCR amplicons. For zebrafish, 2 nl of the injection solution containing 200 pg of circular reporter constructs and 0.03%/μl of phenol red solution (Sigma-Aldrich) in Danieau’s water was injected into the zygotes. When introducing BACs into zebrafish, 250 pg of circular BAC plasmids in 4 nl of injection solution was used. Procedures for zebrafish were approved by the Academia Sinica Institutional Animal Care and Use Committee (protocol ID: 22-06-1877). Reporter gene expression was observed and imaged with either a Zeiss Axio Observer Z1 or a Nikon SMZ18 microscope.

### Statistical analyses

Results of each transgenesis experiment in hemichordates, sea urchins, and zebrafish are listed in the tables S1 to S3, S5, and S6. Statistical comparisons in activities of different CRMs or BACs were performed using two-tailed Welch’s *t* test with 95% confidence intervals. Bonferroni correction was applied to control type I error rate for multiple comparisons, and *P* values are shown in Figs. 3 and 4. Statistical graphs were generated using GraphPad Prism 10.

### Whole-mount in situ hybridization

Antisense RNA probes were synthesized following the instructions of DIG RNA Labeling mix (Roche) or LabelIT DNP Labeling kit (Mirus) to generate respective DIG-labeled or DNP-labeled probes. In situ hybridization of *P. flava* and sea urchin embryos was conducted as described (*34*). *A. typicus* in situ hybridization was performed according to the same protocol as used for sea urchins. Single and double fluorescent in situ hybridization for sea urchin, *P. flava*, and zebrafish was performed following the previously described procedure (*83*–*86*), except that 100 mM sodium azide was used to quench endogenous peroxidase activity or antibody activity. Tyramide signal amplification (PerkinElmer) was applied to amplify the fluorescent signals. Embryos were imaged with either a Zeiss Axio Imager A2 or a Zeiss Axio Observer Z1 microscope. Fluorescent signals were captured using a Zeiss LSM 880 confocal microscope.
