# Supplementary material for: Deep homology of a brachyury cis-regulatory syntax and the evolutionary origin of the notochord
Source: Sci Adv. 2025 Jul 25;11(30):eadw3307. doi: 10.1126/sciadv.adw3307 (PMC12292651; doi:10.1126/sciadv.adw3307)
Supplement: Supplementary file 1 — Figs. S1 to S8 Tables S1 to S8 [file sciadv.adw3307_sm.pdf]

Science Advances
AAAS

Supplementary Materials for

Deep homology of a *brachyury* cis-regulatory syntax and the evolutionary origin of the notochord

Tzu-Pei Fan *et al.*

Corresponding author: Yi-Hsien Su, yhsu@gate.sinica.edu.tw

Sci. Adv. **11**, eadw3307 (2025)
DOI: 10.1126/sciadv.adw3307

This PDF file includes:

Figs. S1 to S8
Tables S1 to S8

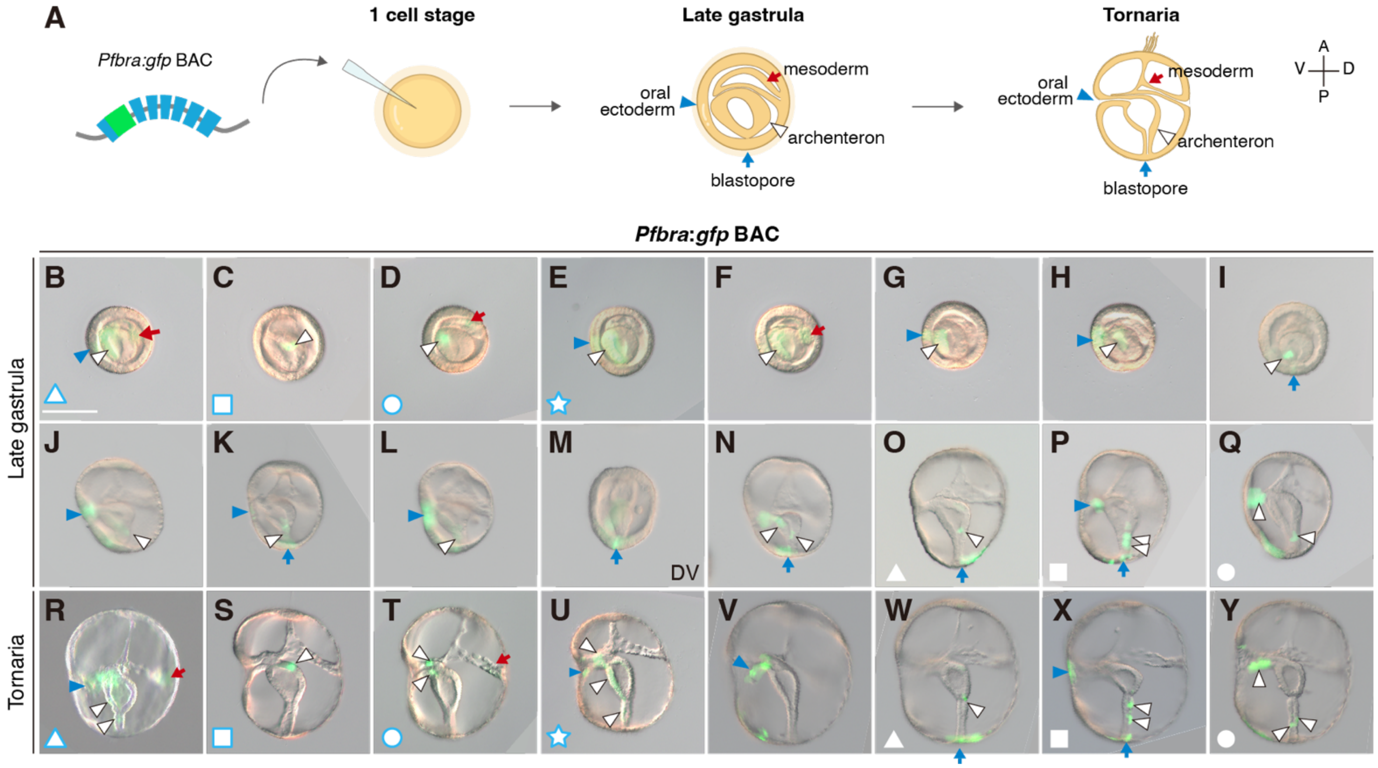

Fig. S1. *Pfbra:gfp* BAC recapitulates the endogenous expression pattern of *Pfbra*. (**A**) *P. flava* zygotes injected with linearized *Pfbra*:*gfp* BAC were observed at the late gastrula and tornaria stages (A, anterior; P, posterior; V, ventral; D, dorsal). (**B-Y**) Images of embryos from 5 independent experiments (table S1). The same embryos observed at different developmental stages are marked with the same symbols in the bottom left corners. On average, 30.1% of the injected embryos showed GFP signals, which were observed mainly in the oral ectoderm (blue arrowhead), archenteron (white arrowhead), and blastopore (blue arrow) at both late gastrula (B-Q, 38-48 hpf) and tornaria (R-Y, 72 hpf) stages. Ectopic expression in the mesoderm (17.6%) is indicated by red arrows. Embryos are viewed from the lateral side with the mouth to the left, unless otherwise indicated (DV, dorsal view, in panel m). All panels are shown at the same scale, according to the scale bar (100 μm) in panel B.

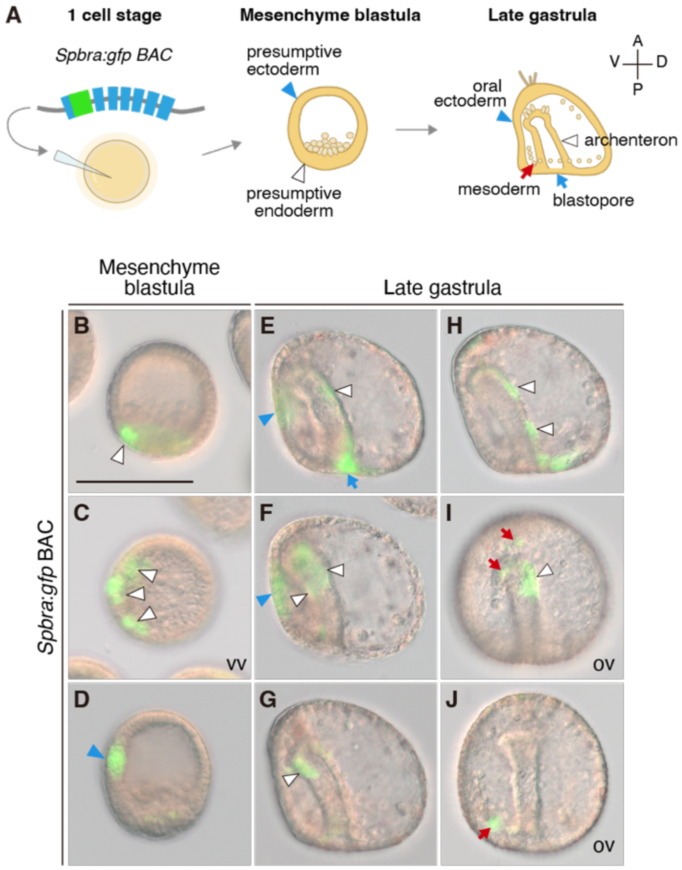

Fig. S2. *Spbra:gfp* BAC recapitulated the endogenous expression pattern of *Spbra*. (**A**) *S. purpuratus* zygotes injected with linearized *Spbra:gfp* BAC were observed at the mesenchyme blastula and the late gastrula stages (A, anterior; P, posterior; V, ventral; D, dorsal) (table S1). (**B-D**) GFP signals were observed in the presumptive endoderm (white arrowhead) and ectoderm (blue arrowhead) at the mesenchyme blastula stage. Panel C shows the vegetal view of the embryo in panel B, with GFP signals present on one side of the embryo. (**E-J**) At the late gastrula stage, GFP signals were observed in the oral ectoderm (blue arrowhead), blastopore (blue arrow), and archenteron (white arrowhead). Ectopic expression was also observed in the mesodermal cells (red arrow). Unless otherwise indicated, embryos are viewed from the lateral side (VV, vegetal view; OV, oral view). All panels are shown at the same scale, according to the scale bar (100 μm) in panel B.

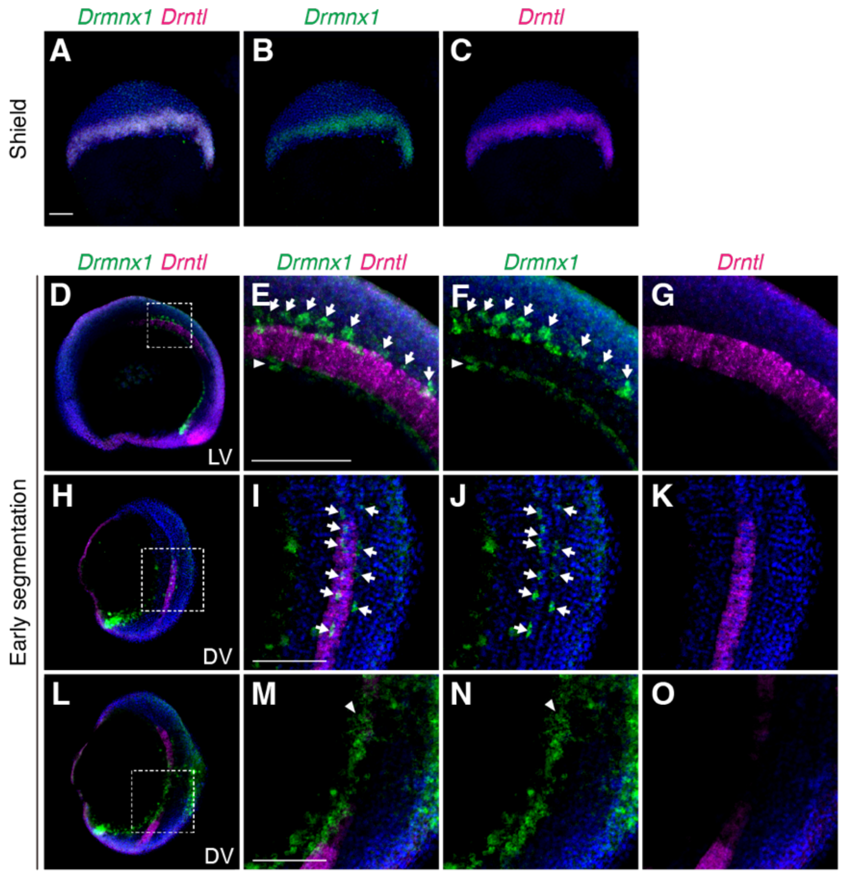

Fig. S3. Expression of *Drmnx1* in motor neurons and hypochord of zebrafish embryos. Double FISH of *Drmnx1* (green) and *Drntl* (magenta) in zebrafish embryos. Nuclei were counterstained with Hoechst 33342 (blue). (**A-C**) *Drmnx1 and Drntl* transcripts were detected in the germ ring at the shield stage. (**D-O**) *Drmnx1* expression in the motor neurons and hypochord at the early segmentation stage. Panel D is a lateral view with dorsal side to the right. Panels H and L show the same embryo (dorsal side to the right), with different Z-stacks focusing respectively above and underneath the notochord. The white dashed boxes in panels D, H, and L are magnified and split into single channels shown in panels E-G, I-K, and M-O, respectively. *Drmnx1* expression in the motor neurons (white arrow) and hypochord (white arrowhead) is indicated. All scale bars represent 100 µm. Panels A-D, H, and L are in the same scale, and panels E-G, I-K, and M-O are in the same scale.

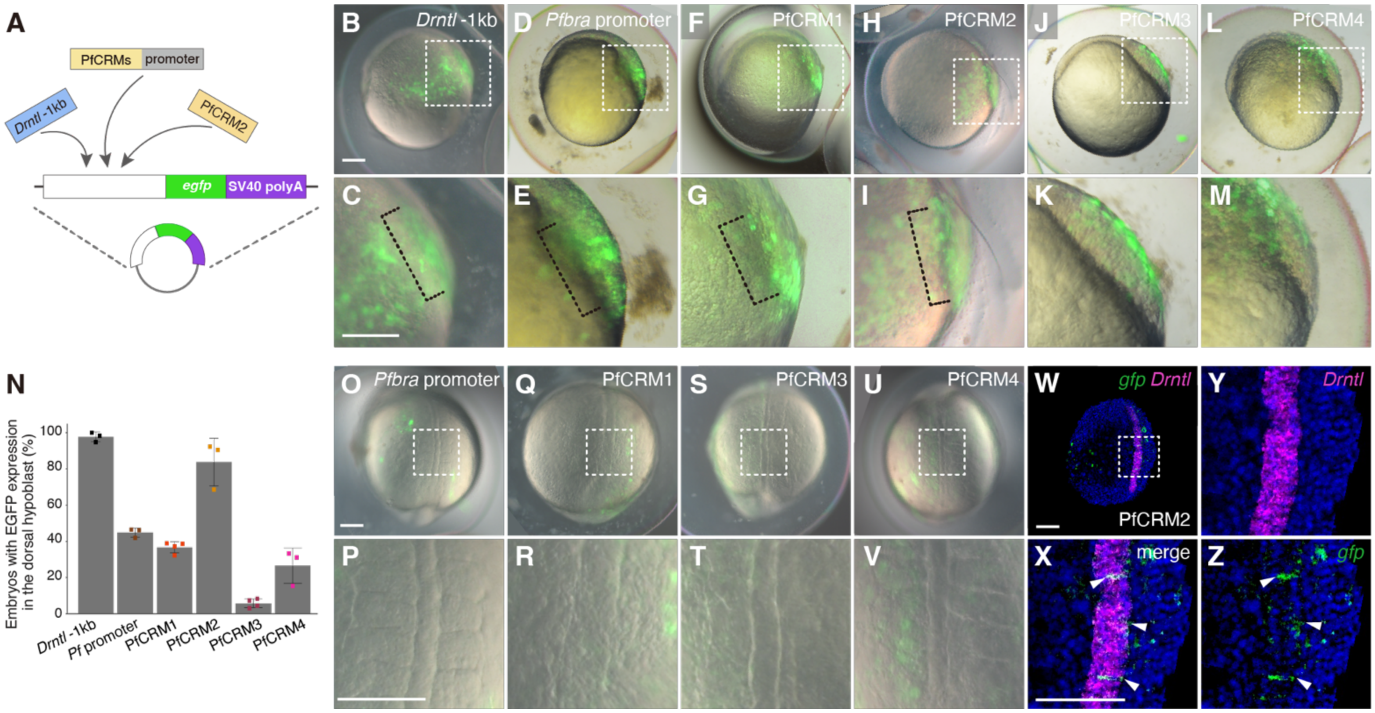

Fig. S4. Transcriptional activities of zebrafish and hemichordate reporter constructs in zebrafish embryos. (**A**), The illustration depicts structures of the reporter constructs. (**B-M**) Activities of the *Drntl* notochord enhancer, *Pfbra* promoter, and PfCRMs in zebrafish embryos at the shield stage. Embryos are displayed in lateral view with dorsal to the right. Magnified views of the shield regions (white squares) are displayed in the lower panels. Black brackets highlight EGFP signals in the dorsal hypoblast (a layer of involuting cells that primarily give rise to the notochord). Embryos in panels K and M exhibit EGFP signals in the dorsal epiblast (cells that contribute to both notochord and the floor plate), but not in the hypoblast. (**N**) The chart displays percentages of embryos exhibiting EGFP signals in the dorsal hypoblast out of the total number of EGFP-positive embryos. Each data point (colored square) is the result of a single experiment. The gray columns are average results from at least three biological replicates, with error bars showing standard deviations. (**O-V**) Activities of *Pfbra* promoter and PfCRMs in zebrafish embryos at the early segmentation stage. Embryos are shown in the dorsal view. The notochord regions of each embryo are enlarged and displayed below the respective image. (**W-Z**) Double FISH of *Drntl* (magenta) and *egfp* (green) in zebrafish embryos injected with the PfCRM2 reporter. Nuclei were counterstained with Hoechst 33342 (blue). The early segmentation stage embryo is oriented in the dorsal view (slightly toward the right side). The notochord region in panel W (white square) is enlarged (X) and split into single channels (Y-Z). White arrowheads indicate *egfp* expression in notochord cells. Upper panels of B-M, lower panels of B-M, panels O, Q, S and U, panels P, R, T and V, and panels X-Z are in the same scale, respectively. All scale bars represent 100 μm.

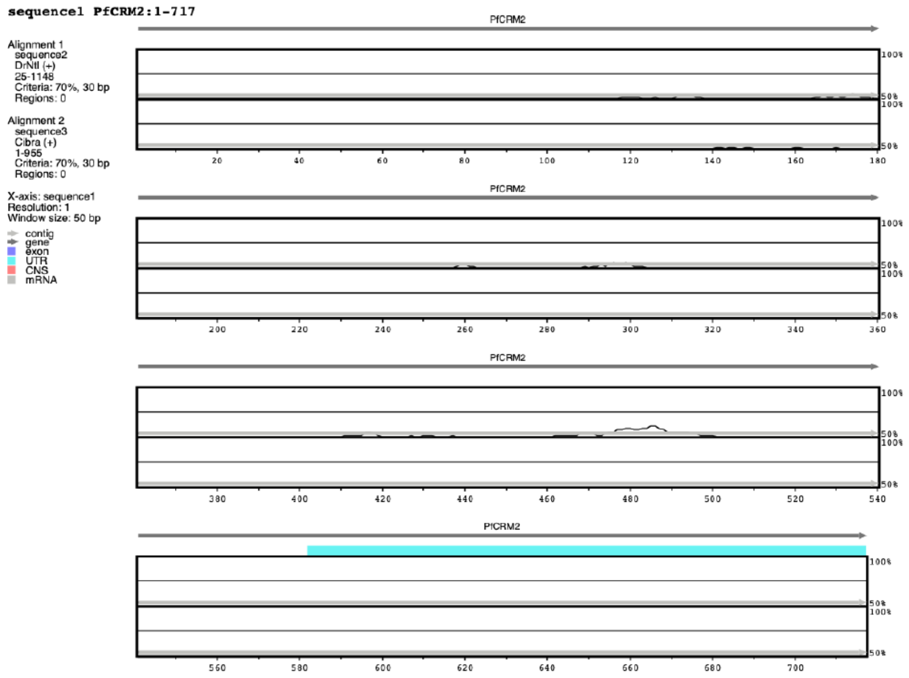

Fig S5. No conserved regions are detected between PfCRM2 and the notochord enhancers of zebrafish and *Ciona*. Sequence comparison using mVISTA with PfCRM2 as a query to align with the known notochord enhancers of zebrafish *Drntl* and *Ciona Cibra*. The criterion of 70% conservation in a 50 bp sliding window was used. Regions with 50-100% identity are displayed. The conserved regions should be marked with red peaks, but none are observed in the plot.

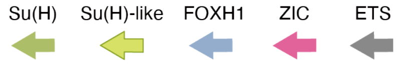

***DrNtl* -1 kb**

```
-1148 CAAGCTCACAAGGTTAATTTGAGTCAGCTTAAAAAATTGAATGCAACCAGGATGCTTTTACAGTGTATGTTTACATTGTAATGCATTTGCAGAAATGAAA
-1048 TCGAACATTTTAATTGTAGCCTTTTTGGTATTAGTTATTTTTAGAGCTGTAGCTTCTAATCCGTCTGCGTGTTAATAAAAAGCGCATTAATAGCTATGTC
- 948 AGGTCGTTCTGCAGGACGCTCATCAGGCTGGAGTCATGCTGGATTAAGGAAATTCACACAGATCGTCACCTCTGGCCATCCACACTTCAGTCGCGTCAAT
- 848 GGTCGCATCCTAATGGCCGTCCCAGTAAACACGGGCTTTCTCACATGTGTATCTGGGACAACAAAAGATTAGCATTATTCGCAAATATACAACTGAGTTC
- 748 AAAGGCAACGAGGCAGCAGACACCGTCTCTCTTCCTTCTTTTGTTTCAAAGACGAGCTCGCGAAGAGCAAAAACATGTTTGGGTTTAGGCTTTCATTCCA
- 648 CAAATATCCAGTAAACAACCGGTGCTCTTATTAAATGAGATGTGTTTAATGAAAAATATTTGTAGTAATAATACAAATATATCAAATGCAAATGTATTTA
- 548 AACCCAGTAGMTTAGTTCTGGTTTAATAAAGGAGAACCAATAAAAAGTAGGCAAGAAATAAAGTCTATGTCTAATGAAGTCTAAAGTCTAATTAAATAAA
- 448 ATGTGAACTATTAACTTGCATAAATTAAACATTATTTAAAAAAACAATCTTGGCATGCACGTTATTTTAGGCCATTTTTAATTATCCATAGGCCTACTTT
- 348 TTATTTTCATTTAATTTAATTTTTTTATTTAGGTAGACCTGTTTGTTTTTATTTTGTGTTAGTTCTTTTCACTGACATTCACCTCAACACTTTGTAAATG
- 248 GGCCACTTTGTACGTCAAGAATACTTTCTGTTTTGTGGCGTCAAATTATCGGGCGCTATTAGGGCGCGATGGGCCTGGTCTGGCGCTCCCGGTCACGCCC
- 148 CGTAACAGCGGGAGCTTCGTATCGGGCCTTTAAAAAGGCACCGGACAAGTTAAAAAAGACCAGATTGAGACGCGCTGTCAAAGCAACAGTATCCAACGGG
-  48 ATTTAGTAGGATCGTCGGACTTATCTCAAGCTTTATTTGATCGGAAAT
```

***Cibra* SFZE**

```
-792 GGCAATTAGTTTCCTTGTTTACGGAACAAACGACTGCGGGGTGCGAATTGTGACGTCATCAATCAAACTAAAAAGGGGAGTTGTGACGCAATAATAAAG
-782 TAATTTTCAAATGCGATTGTTATGTAATAAAAAAGGAAAAAATAATGAACATAATTATTCGTTTCTTCGACTGCGACAGCGAAGATAAAATTAATTAAAA
-772 CAACAAAGAGAACGAAATTTGCAACAAGACGACAGTCGATAAAACACGATGAGTAGAAACTACTTGAGTAAAAGTGTCAAATAAAACAAAAAATGAAAAA
-762 AACACACCCAACGTACAATAAAACTTACGGCAAATTGTAGTTAAAATTCATAATTACAAAACAATAAAGATCATATAAACAAAATATTAAAGATCATATT
-752 AACAATATAATATAGATCATATTAATAGCGACAAACCTTATCTGGTGTTACGTCACAATACAAACAAAATATTTTGACATGTCAATCAAAATCGGAAACC
-742 AAGTTTCAACTTCCCACGCAAGACAATGGGAAAGTAACACGTCACAATACACTTGGTGACGTC
```

PfCRM1

```
-3454 GCAGAAAATCAACACGTGAGTCAGGTATTACTATTTGAAATCTAAGATAACGGACTTTATTTTGTCAAGATTTTGCGCCAGCGAGGTTTGATTAGAACCG
-3354 ATTCACACTTTTGATGCTCTACCCATTCATAGTTGCCGTTGACGGTTTCCCACGAAAAATCAAGATTATGATCAAAGTTGAGACAAAAAAACACGAGAAG
-3254 CAGTGAACTGATTTCTTTTATACGGGGAACAAGTCAAAAGTATCCGCTAACGGCTTTAAAGAAAAGGAGATACCCGAATCTGTCGTGCCGCTTCGACATA
-3154 CCCGCTAACTGCTCTACAAAGGTGAAAAATGTAACAACTCCTTCGCGTAGTGTATGGAGTGGCCATAGCACTGTGACTGTGCAAGCCGAAAATAGAGTGA
-3054 CTACATTCCAACTAAATTGATTTTTTCATGATCGGCTTTATGAAC
```

PfCRM2

```
-717 CAGTTCCAGCCCGAGTTACATGTTTTTATTATAGGCGCCAAATTATGAGAGAATACTATGGATATAAGAGCATGTTTCAGCTGTTGCAAAAGCAAAAAAT
-617 GTCACTAACCGTTAATCGCTACTGCAAAGCAATACAATTGAAAACTTTTCTTGCTGCATACAATGCATGAAGAACTTGTCAAATTCTTACAACTTGTCTA
-517 GCTATATTCTGCACGAGTTAGTCAAACTACCCAGCCCTACCGTTCATTTGGCCCATGTGCAATCCTATGTACATGACAAATATTGCTAAGGAATTCCCAC
-417 GTCAAGAAAAAAAGGTGTGAAAACCGATCGGATAAGACTCTGGCGTTAGGGCAATTAAATTGGGAATTGAAACCGATAAATGTTCTGTGCAGCCAATAGG
-317 AGCGCCGCATTCGCTCATGAATATTGACATCATCGGATCAGCGGCGAAACTCAGAGAGTACTTTTATGTTATTTGCATATTCATTAGGCGGCTCACTTTA
-217 TGTTCCTGATGCGAGTGGGTATCAACACAAACATTTATTCATATGTTGGTATACATAAAAGGTTGGGTCATTCAACACAGTACATCATATCCACATAGAG
-117 CCCTGACTTGTGAACTGTACCTACCAAGCGTGTCAGCACCTACGTCTCTATATATCGCGTGATTGCCAAGTTCAACAACACATCGTGACTTGTTCCTTCT
-  17 CTGTCTCCAGACCTAGC
```

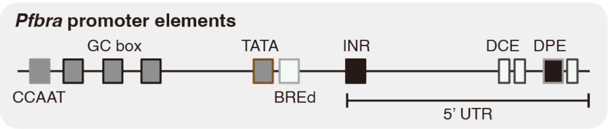

PfCRM3

```
3793 TGACTGGCCACTTGGCAGATCTTTGTCCATTCTCGGCTTAAGTGATAGTGCACTGCTCAGAAGTTTCCCGATTGTAATCGAGAAGATGGCCGAGGTGACA
3893 TCAGTTTGGCTTCAAAGTGTCAATCTGTTTTTGTTGTTTTTTTGTTATTGTCGCCCGTGTCTATCAAAATCTCTGTTAATTGTTGTCTTTGATCAAGTCA
3993 ACGCAGTGACCAAATTCCCGCTTTGACCGACGATCACTCGTCTTTTGTGTCTCAGTTGTCAGGGAGGGCACAAAAAACTCATCGCCGCAGCTGTGTTTGG
4093 AGATCGTCTTTGCTACACTTTC
```

PfCRM4

```
6567 ACATCGGGAAAGCTATGAGTAGCACTTCTTTCAGGTTGGTAGGTTCTTTGATCAATTTTCCTACTGTAAATTGTAAATTAAATATGTATATTTTTATTTC
6667 AATTTCAGCAAACGGGTATCCGTCGGACTCTATGCTGTCGGCGCACGAAAACTGGTCAGGTATACCTGTGACGTCACACACGACCCACACGGCGCCCCAG
6767 TACCCAGCCATGTGGCCCGTCAGCAACGCCCTAACGCCAGTCACCACAGCCAGCAACCCCATACCCACGGGAATAATCCGTGCTCCTTACGCTACCATCC
6867 CGGCTTCCACGGGCAGCCTGCATCATACCGGACACTCTCCAGTCTACGAGAACGCCAGTTCGGATATCTCAGTGGCCGACGCCTTTGCAGCACATCACAC
6967 AGCGACGGCGTCAAAGGCTAATGGCTGGAGTCCCCTGACGCCACCTTCAGTTTAG
```

SpCRM4

```
8201 TGTCGCCTCTCTCTTTTTCAAAACATATCCTATAAAGGAATCGGAGAGGATAAAATAGATCTCTTTACACCTCTCATAAAGTGATCAATTCTTTGATCTT
8301 CTTCTTTGCCGCTCCGATTTGTATTCAGACCCTATAAAATATTTCTTCCGCACTCTAAATGAACGCACTGGTAATAAAACGTCAATCGTCTTTTTGGAAG
8401 TTTGTGGTATACATCGGACAAACCGCCCTTTCTTCTTCTTGGTTTGTTTTTTATAAGCACTCGTGTTTGAAAATAAGATGAATAGAAATAGAGATCCCCG
8501 ATTGAACTTACGTTTAGGGATAGGCGATGGGTCATAAAAAAGGGAGATTATTCACAAAAAAGGGAGATTATTTCGAGGCGTTTAGAAGTCTGGTGTTGAT
8601 CCGCGCAACTTTAATCGCGTCAATAGGATCAATCGATCGCACAATGCGTCGCGCAGTTAAGCGGACTGTTGAATGTCTTTATGGACCAAAAATAGAGGCT
8701 TTGCGATGAGTCGTAGATTGTTGTAGTTCTGGATGAATGTTAATTTGTCCCTTTTAAGAATCTGTCTTGAAACAATTTCATGACCTGAGTTAAGTAAACT
8801 AATCCGAACACAAATTTAAGTGGATTACGCAAGAATCACACATG
```

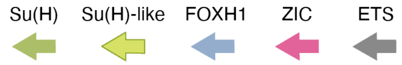

Spbra promoter

```
-977 GAACAGTTGACCAATCACGCGGTGGGTCTCTGCAAAGGTAGGCACACTCGTCGCGTACTCGACAAGATTGCACATACAAAACCGCTTCTGATTGGCAAAG
-877 GCGGCTCCCTTTGATCTCGCAGCTCGTCCACATGCAGCACAGCCGGTATCATGTCAATCAAGCCGCCAAAGCCACGCCTTATTTTGTCACTTCTAATGGA
-777 TACCCTCCACGGCGAATCCTGAAGAAGAGAAAAGCCAGGGTGGTCGAAGTTTTGTCAATAAAAGCCCACCAAAAGTCCTTTTCATTCGTGATCGCCGAAT
-677 AAACATTCGCACTTGCGACGTGAAACGAGACTTACGCCGGAGAGAAAAGGAGAAAGTGACGAAAGCACCACCCGGTGTTTAATCTGGTAGTTCTACCAAT
-577 CTTTTGACTTAAGTTGACTTAGAACTTTTACAAGCAGGCGATCGACACCAGAGAGAGTTCAGTAGGACCAGATTGCGACCGATCCGCATAAGACCAGTGA
-477 CTCACACCACTTTTCAAGACGTGTTCACTCAATCGAATTTTATGAATCCATTCAGACTCGGCGACCCTTCGACACAGCTCGTATAAATTGTCGGCGGTAC
-377 CTCAAAACTGAACACATTCAATCGTCTGCGAGTGATCGCCGTATATCGTCCATTTAAATGGTTCTGGAGGTTTTATAAAACGGTGTTTAACAGTCCCTCG
-277 GGATCACTTAGTAACTATTTCGTGAATCTTTATTTTTTTCTCAAACTTTGCTTTCGTCAGGCACTTGCATTTCGGTTTGCAAGTTTTATTTTCTTCGTGT
-177 AACACGACATTGTCATACTTCGGAACATCCTGATTCGATGTCACTTTGAATTTACAACACGGGAGAACTTCTAGCTGCTGGAGATTTTTGTTATATTAAC
 -77 TTTATCCCAGCGAAAAGCGAAATCGACTGCTGCCTGTCCGAGCGCCCGCGGAAAACTCTCAACCCATCAACGCCACT
```

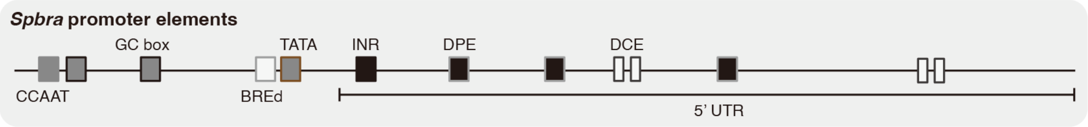

DmCRM1

```
-5126 TAGTTTAAGGGTTTTTGTTAGTGAATATCTTGATCTTTGTATTGCATCTGATATTGGTTGATATCAACCACTTATTAAACTATTTACATATGGACTCCA
-5026 AATAGTAACTCAAAAACCATGATTATGTACAGTAGGCATTTAATGAAAGAAGTTTCAATAACAACGGATGGGATATAAAGATAAAAGATTCCTTCAACTT
-4926 CCAATCTACGGCTGAGACAAGGATTCCTTTGACAAATTCAGAGCGTCTATAATAAAGATCACAAAAGATAAGAGACTTTTTCTCTCTGTGCAACAAATGT
-4826 GAGCAACTGAAACACGTTACACTTAATTTTTGGCGCATGCAAATTGAACGATTAGCCACGGGGGTTACAGCAGGGGTAACGTATAAATCCCGCCTAGGAC
-4726 TCCAATTCTGGGCCTTCTGAGAGTTAAAGGTGTGCTTGCCCTGAACGATCCGACCCACCTGAGAGTTCGATGTTATGGGGGCTCTCTACCTGTTGCCGGG
-4626 TTCTCGATGGCGATGGCCGCAAATTGACCAGCCGGGTGAGCGAGCGAGAGGCGAAATACCGCCGCTGTCTTACCTGTTAGAGGGTGTGGCCTAACCATCC
-4526 GTCAGCCAGCCGATCATCGATCGGGCCAAAACGAGATGGCTATAGGACCGGTAGAGAGCTAGCACGCTAGCCGGCCATCATATCATCCATGGGCCAAAAC
-4426 AAGAAAACAACAACGATGCCACGATGTCGATCGGCTGGCCAAAGACGAGAGATGAGAAATAGAGGAACCGAAAGCGCGGCTTGTGATGGTTGAAAAAATT
-4326 AGCACCGATCGTTGATCATTAGCTCGGGAACGGTGGCAACGCAAACACACGATCGGCGGCAGCGCTAAACTCCAATCTCGAATCTCGACTCGAATCAAAT
-4226 ATCTTGAAATCTTTGAAAACAAATTTCACGCGGCTGTATCTTCCCACTTGAATCTGAACTCATACGATAGTGATCCTTGATAAAGTGAACCCTCTAGGGA
-4126 ATTAGTGCTATAAAAGTGCGTGATTTGGCAGTGTTGCGTATTCAACTAAAATCTAAATCTAAACCTAAACCATCTTCATAAGGAGTTTACCACTGGATTA
-4026 TATCGAGAGCGCCGAGTTTCGGCGTGAAGCAGGTTACCAAATTGGTAAGTTAGTTGCACTTTATCTCAATTTTGGTTTCA
```

NvCRM1

```
-5772 CCCTTGTCTGCGTTCAATATCTGACGACAAGGAATAGGGCAGTGCTGAAGATAAGGATGTCTAGTACCAGTATGCCGAATTAGGTGGAGCTACATGCAAA
-5672 TGTTAAACTACGTGCTACTGCTACTTTAATTCTGCGAGCATTTAATCAATGACCGTGCTATTCTGGAGACCCCAGGTCCGTTTTTCGGTGTCCGTCATAG
-5572 CATGTGTAATGTCCGTTGCTATCGGTCGTTTTGAATTGATGTCATCAGTGATGAACAGCTCTCGCGATAAGCGCCTAGCTTCGAATAAGCGCAAAAAATT
-5472 AATTAAGCTCCCACTATGATGAGCTCCCCCTAAAGAAGAATGTAAGAATGACTAGACGCAATGTGTTTAGCGCCAAGTCTTTTTCTATAATTGAGCAACA
-5372 AAAGACGTGTACTGGACTTTTTGTTGGATTTACTGTTTGCTCAATTTCTTACTATTCGATTTTTTTTTCAAATACTTGAAATGAAGAAATGACAATTTTT
-5272 AGAGTTTAAGCCTATTTTCATGATTATGATTCATATCGTCTTCGAATGATCAGTAGCGGATCTAGGGGGAGGGTTTAGGGGGTTTAAACCCTCCCCCCCC
-5172 CCCCCCCTTGGGCTGCCAGAAAGCCATATGCAACAAAAAAATTACACCCTTTCCTTTAGTCGCTGAGCCAAACCCTCCCTTCAGCGAAAAGCTAGATCCG
-5072 CCCCTAATGATTATAGGGGAGGGTAGGCATATTAGGACAAAACTTTGGCATCGAATCCGTGGCCGCTACCACAGTGAGCCACTCGTCCTCTCAC
```

NvCRM2

```
-2661 TTGCGGAATTGATATTATTAGAACTATGGAGAGAATTCTTAGGCTCTCTCTTGTTTAATTTGATAGTATCTTATAATGTCCTTGTATGCCACAGTGCAAA
-2561 ATACTGCGCACGATTGGAAACTAATTTGTGTTATCGTTGCCACTTAAATTGTGTACACCTCGTAAAACTCGATGACACGAGTCAGTTCTCTGTGGTTTTT
-2461 ACTATCTCCACATGGCGAAATGATGTTAAGGTGTTAATCATTTGGAGGTTTCATCTCATTTCCCAGCTTGTCCGCGATGTACGAGTTCGACTTGCTGGAT
-2361 TGATTTGTTTGCTATGAAGGTTGGAACGTACTCGCGGCACGTAGCTCTATTGTTTTCTATCGGAACTGGACTTGCCTCTGTATGTTACCGCATTTTTAAT
-2261 ATCCATGGAAATTGAGCGTGGAATTTTTAGGGCTCTTATTAATCAAGTGCGGTGGGATTAATATGGGTGGATCGGAAATTCTTTTTACGAGCTATAAAGA
-2161 CACAAAATAGGCTCTAAACTGAACTAATCCTATCAAGTATGTGATTATTTGTATTCGATTCGTGAACAAGTGAAACTCTGCAAAAAAAAAAACTCCCTAC
-2061 AAGCAAACTAGAATACATTGAACTTTATTGACATCTATTCTGGAATTCAACATGTTTCCGAGGTTATGAATTATTTTGATA
```

NvCRM3

```
-1499 TCGGTGAAATTGCTGTTTGAAATGCCAGGCGTAAAGGCTCGCCCACTTGTTTGCGCAAAATCCGTAATCTGGAATCCGACGTCAAGCTGTCACGGATGTC
-1399 TCTGTGATGCCGCCGAATAAGCGAGCTGGTTTCCAGGTAGCGTTGGGAACAAGCGACTAATGGAAGGAAGCCCACTAGAACTAATTGCTTGAGTAAAAAA
-1299 TCGAGTTATCTTGGGGTAATAGTTCTCTCTGGCCAGGTATAGACCATTCACGCTGTTGGTGTGTTGGTTTGTTTATGGTAATTTCCAACGCCGGCCCCTT
-1199 TGAAGGGCTAGCTTAAACGCTACAAGTGAATGGTTTCATCCCCTGTCGGGCAACCGTACCCGAGAATCAATAACACGCTAAGTCCTAGTATTGTTTTGCG
-1099 CTTCCACACAAACATTGTGTTGAAATTAGCATCAACAAAACGATTGCGATGGAAATAAGTGGTGATTGTGCATGAAGAAAACAGGCCGCGGTGGCCGCTC
- 999 GTACCGACAAACGCAGTCGGCCAGGAGCTAAGTTATTGGTTTTGTCGGCGCGTTTGCTTCACAGCTAGGTCCAGGAAAAAGGCGGTGGACATTTTTTTAA
- 899 TCGTAGTTCTTATTGCGGCCTATCACTTATTACACCCATAGCTGTGAACCATAACCGTAAAAAATGTGTGCACAGATTTTAGCCGTAAAGATTCTAATCT
- 799 TCGCACGGAGCATTATACAAAGTGACACTCTTCTATTTGGTCAACGACCTTTGACTACAAAATGTTAGAGTTGGGCGAGAGGATCTGACTGACATGTAGA
- 699 TACTAAAAAAGCGTTAGATTGCCAGTTTTAATAATATAAGTACTTTAAGTATAACCCTTAATTAAACTTATTGCTAGATACGATGACGATTTATGTCACC
- 599 GCGGTAAAACTACGATGGAAAACAATAACTTCCAACGTGTGTTAATTTTGGTCCGCTGCTACGGCCGGAGCCCGATGCCTCGACCTCTATCATGCACGCC
- 499 CTTGCTAAACGTCCTTGTTAAATGGTTTGACCCAAAAGGTTAACCGTCAGTAAAATTGTGTCAGGTTTGTTGGCTTATTATTTCTCACCCTATGAGAGAT
```

Su(H) Su(H)-like FOXH1 ZIC ETS

CoCRM4

6709 TCAAAGTCGACAATGGTGTCGACCGAGCACTCGAGCTCCTCGCTCAGCAGCTTGAGGAAGGTCGAGTGGTGCTTGCCTTCGACCCCGGCCCTGCGCTTGC
6809 CGTCGTCGCCCTTGGTCTTTTCGAGACGGGACTTGACGGCGGTGGCGAGCATCGGGACGAGCTGCGTCTCAGTGTTGAACTTGAAGCCGTCCGAGTTGAC
6909 CTCGCGGTTGAGGTGAATGGCGAGGGTCGGCACACGCAGAATCGGGCGCTTGATGTGCACGAGACGGTGCTGCAGCTGGCCGTCAACGGCGCGGACAATC
7009 GCACGACCGGCGACAGTCAGGTCGCGATCAAACCAGGTGTGCCACAGACCACCGCCGTAGCATTCGACACCCAGCGACACATAGCCGGCCTTGGACAGCT
7109 CGCTCACGGGCTTGACACGCAGGCACGGGCTGTCCGTGTGCGCACCGACAATCGAAAATCCGCTTTTGGTTCCAGGCACAAACTTGCCGCCGACCGTGAA
7209 TGCGATCAGCGTCGACCGGTTGCGCGTGAAAAAGTACTGTCGGCAACCAGAACCAACAAAACACAAGTATGGTTGGGAATTTGAGCAAAACAAAAGCAGA
7309 TTGTAAATCGTAACACAAGTGAGCACTTTTGTATCTCATATGCTGAGATTGACACGAGCGCCCAATGTAATACAATGGGATCCCTGACCTTGCTGTCGGG
7409 CTTCAGCGACCATGCTTCCTTCTCCTTGAGCTCAATGAACGACGATGCGAGCAGTGTGCGGCGGCACTCCTCGACGGCTACAATGGAGGGGGAAGCCAGC
7509 GCAAGACAAGGCAGGTAGGTGAGTGAAGTGAAGTGAAGTGAAGTGAATGCAGGATGCACACGTCAATCGCAAGCAAGAGTGAAGAAGAGCATGCATGGCG
7609 TGAAGCAGAGTTGCGTACCATGGAATGGAGATGGGCTCGCATTCACAAAGGCGAGGAAGCGCTGGGCGAGTTGATGGGCGTCGCTGGTCATTGTTCGGAG
7709 CATGTAGC

***Bfbra1* SFZE**

7657 TGTACGGGCGGTCACCCCTACACCGCCGAGCGCCTCGATGGTATGGTTCCCACACACAGTCGTGCAAATGTCGTGACAAACTCGTCTCTTTGATATTCCC
7757 CGAACCAGCACGCAGTACGACATATGTCGGCATGACTTCACAAGTCCATGTGGAGCGTGGTTCACAATGGGCCGGACTTGTTCATGCATTATTACAAGGT
7857 GCGAGGCGGCTCATCCCTTTGTTCTATCATGTCCCTGCCTTCTCTCCATTGTTCTACCATTCGTTTCATTCGCACAAGTCTTTGCCATGCATAGGTGTAG
7957 ATGTCCTCTCTAGAGACGTCAGACTTGATATTGACATCTCTGGTAGGTTTCCCAGGGGAGCATCTTTCTACACGGTTGATGTTCTAATCAATTAGCAGTG
8057 TCTAATTAAGTCCCCCGCTGCCTTCCGTGTACCCAGCTTCCTCGCTAATCGACTGTATATAAAAGAAGTTGT

***Bfbra2* SFZE**

4973 TGTGATTCAAATTCTACCATATCTAAAAAGTGGGAAGTTGAATTGATTATCTAATGTTAGCTGGTCTAAGTTGTATGCTAGCCAACTTTTAACATGCCTG
5073 TGTGTGCTTGTGCCCCGCACAATTAAAATGTGCAATGGTCTTCATTCACTTATTTTCTTACTCACTGTCTCACTCACTCACTATATATGTAATTTTTTAT

\\

6573 GGAAATCTGCTAAGACGAATATACACAAGTAAGCAAGAAACCATAATATCAAACAATCATATCATTTCATTTGAAGAAATCCCACTCCTCAGAAATATTG
6673 AAATGTCGCTGCTATTTTGAAGCCCATGTCGCAGGCTTGTGCCCGACACGCTATCGTCCCTCCGCCAAGTTAGCCGTAAGTTGGTGAGAACTTGTCAGGA

***Drbra* 5' SFZE**

−4775 GACGTTATGCAGAAAATAAGATTAATGCACAGCCTCTCACTCTCTTACATGGCTGACACCCAGAGGTGAGACTTTTACACGTCAGACATTCCATTTAAAT
−4675 GCAGCGTAGGCTGTAATTAGTTACACACTACTAGTAATTGTGATTACTTTTTCAGCGGGAAAGTAATTTACTTTAAAGTAAGGGATTACTTTTCATTTTT
−4575 AGGTCATTTAATGACAGCTACTTATACTTGCTTAAAGGTTTCCTGAAACTGATGTTAGTTTCAGTTTGTTTTAAGGATATCTATTAGCTAGTGTGCTCCA
−4475 AAACAGTGACAAAATGTATTTTTATAAGATATGTTCATTACTTATGCCTAATAGATGAACTATTTTTTACAAGTCATCTCATACTTCAGCTTCTCAATAA
−4375 ATCTTGACCAATCAAATGCTCTCTAGTATCTGACATGCCCCACCCCCTTTCATACGCTTCTCATTTGCTTTGCTTTTGATGCGCTTGTGCTCAACCACTC
−4275 GCACTGGCAGAGCTGTGATAAAACCAAAAGCTACTGGTTGGTTTTTTAAAGGGGAGGAGCTGCTATATGTCCCGCCCTCTCTTCATGTTTCAGTTGAGAT
−4175 TACATCAAATATCGAATAAAAAATGCACATTTCAAAGGACCTTTAAGTACTGTAGGGTCTGCATAAATTCAACAGTTCAATCAATTCAGAGAGGCAGTGT
−4075 GATTGTATTTTTTTGAGTGTGGAGTTTGCATGTTCTCCTTGTATTGGTGTGGGTTTCCTCATGGTGTTCCGGGTTCCCCTACAGTCCAAAAACATGCGCT
−3975 GGGTGAATTGGATAGACTAAATAGGCTGTAGTGAATGAGTGTCAATGTGAGTGTATGGGTGATTCTCAATACTGGGTTGCAGCTGGAAGGGCATCCGCTG

***Drbra* 3' SFZE**

11994 CTGCTGCTGCTGCCGGTGGAGGCTTTATTTACACAGAACTCTCTTAAACTGTTCCGTGCTACTCAAACACACTCACAGAACGACAAAAAAAAACCTACAA
12094 AATCAATATCTTCTTAAAGAAGTTGAAGCCACTTTAAAGCAGGAGAGCGCTAAAATCATGAACGTTTGGTCGCTGCGTGGTTTCCTAGCAACCGGACTTGA
12194 GAAGTTTTGAGAAGAGCACAAGGCTAACATCGCTAACGCGTCGCTGGACAGATGATTTCGGTGTAAAAAGAGCAAAGGTACAGTGTTGTGTGCAGAATTT

***Xbra* 5' SFZE**

−140863 TTTTGCAAAGTACACATTCTCACAAATCCAAAATGGCTATGTCTTTCTTCTCCAAACTACCCAAGTGCAATGCTACACTAAGCCAGCGGTTTTTATGACA
−140763 TTTCAGTAAATCACCTAAAAATATTGCAATTGGTCTCATTTATTAAAAAAATCGACAGCTGCTGTTGTGGAATTGTGCAACAGGATACGGAAGTGCAGGC
−140563 ACACTAATATGATTGGGTGCAGGGAGTTGACTTCTTGATGCTGCTGGTTGCAGGGAGTTGTTGCATAAAATATTGTGTTGAATGCTGTTTAGCGCCTTAG
−140463 CGGTATATTGTTATTGCTGCTAAAGTTATTGCTAACCACAATACTATCTAAAAAAATCAGTGACAAACCATTTAAGTTTTAAAAACGTTATTTGTTTTAT
−140363 TTTGCATATATCATTGGTGGACAGTGCAGGATCGCCGAGATTAGTGCAGGCAAAGGAGGCAGGAGCTTGAACAGTGCGCACTCTGCGCTCGTGATTGAGA
−140263 AGCTGGGCGCGGAATAGTACAGCAGCAACTGAGAAGGACACGTCTTGAAGGACCCCAGCGTGATGGCGCCAAAATGACGTCATCGAATTGCGCCGCCCCC
−140163 GTCTTCACACCCTCTCCTCAGTGGCTGCAGCATGCTAGGGGAGGAAAGAGAGAGACACGGCCCCAAAATGACATCACTGGATTATGCAGCCAAGTCTTCA
−140063 CGCCCTCTTCTTCTCAGTGGCTGCGGCTCGCTGGAGAAGGAGAGAGAGAGCCGACGGTCGCTAGCAGATTATAGTCAGACCCAGCAGAGGAAGTTGGCAA
−139963 GAGGGGAGGGGCGACACACAGTTAGAAGCGCTCGCAAATAAACTGGATTGGGAAGGTATATAAACAACCACATTTTCTTCTATTTTATACAATTTGGAAT
−139863 GTATTTATTGCAAGTAATAAATAATGGTATATGTGCCCTTTAAGTATTTTTATCAGTAATAAAACGTTTTTACACTATTACTGCTCCACATCTTTTTACA
−139763 GCAAGGAATTCTATTTGGAAGCTACACCTCGTGATTTGGAGTTATATGATGATTATATTTTTAAACATTTGTGAGTACCCACCTATTACTGAACACGTGG
−139663 TGTGTAAAGATTAAAATAGGCCCACATTTAAGATTAGTGTCTTTGAGGTAGAGGTGTTGAACTACAACACTCACAATGAAATACTACACTATAAGATCTC

***Xbra* 3' SFZE**

12786 TGTCGATAACGCCAGCCAGCATCTTGTTTGCCAATGCCAATCTTGAATTAAGCATTCTATAAATTATGGAGTTAATTGTATTCAAAAAGTCACGGGTGCA
13786 CAGCTCCAATTATTCCAATAACAGTCCACGAAAAAGCCAGCACTCATGATAAATAATATAACTTGCCTGGGTGCAGTATCAAAAATGTTTCGATATACAA
14786 CCAACAGAAGGGATCCGCACTCACAGGTCTTAGGTAAAAAATAAAGAAGTTTTATTGCAAAGATGTAAGCCTAACGTTTCGGCCTCCTCCGAGGCCTTTC
15786 TCAAAGTGACAAAATGTTTGTTAGAATGCCTTAAATACAAGAATTGGCGGGAAACCAGACATAGAGGAAATGACATATTGACAACATCACCCAAAACGCA

***Mmbra* SFZE**

−1716 GATTTATTCCTAGTTCCAGGAAGTCTCAGGCCTTAGCGTCCCCGAGGGTAGAAAAGGTGAAATCTCCCTTGTATACTTCTGCAAACCCACAACACAAACT
−1616 GGCTGGTGCTCATTGCAGGAGGGTCGGGGTTGGGCGCAAATGAGAGCCTTTTGGTGTCTGTAGAAAGAAGTGTCCCGCTCCTCTAGCCAGAGATTTCTTT
−1516 ATATCCTAGACCACAGAAAAGCAGCCGAACACCCCAGTATTCAGTGGGAAAGGGCAGAGGAGGGTAGTCTAGGGCATCCCACCCCAGAGGTTGGCTCCTG

Fig S6. Sequences of *Pfbra*/*Spbra* promoters and SFZE/SFZE-like-containing CRMs/regions from diverse organisms. Positions of CRMs are labeled on the left side of the sequences, either upstream or downstream of the TSS of the examined *brachyury* orthologs. Binding sites of the four TFs are marked by colored arrows. Previously identified TF binding sites in the notochord enhancers of *DrNtl* and *Cibra* are circled. The sequence of the *Pfbra* putative promoter within PfCRM2 is underlined. The promoter/promoter-proximal elements within PfCRM2 and of *Spbra* promoter are enclosed by rectangles. The illustrations underneath PfCRM2 and *Spbra* promoter sequences depict the arrangements of the deduced promoter elements. BERd: downstream TFIIB recognition element; INR: initiator; DCE: downstream core element; DPE: downstream promoter element.

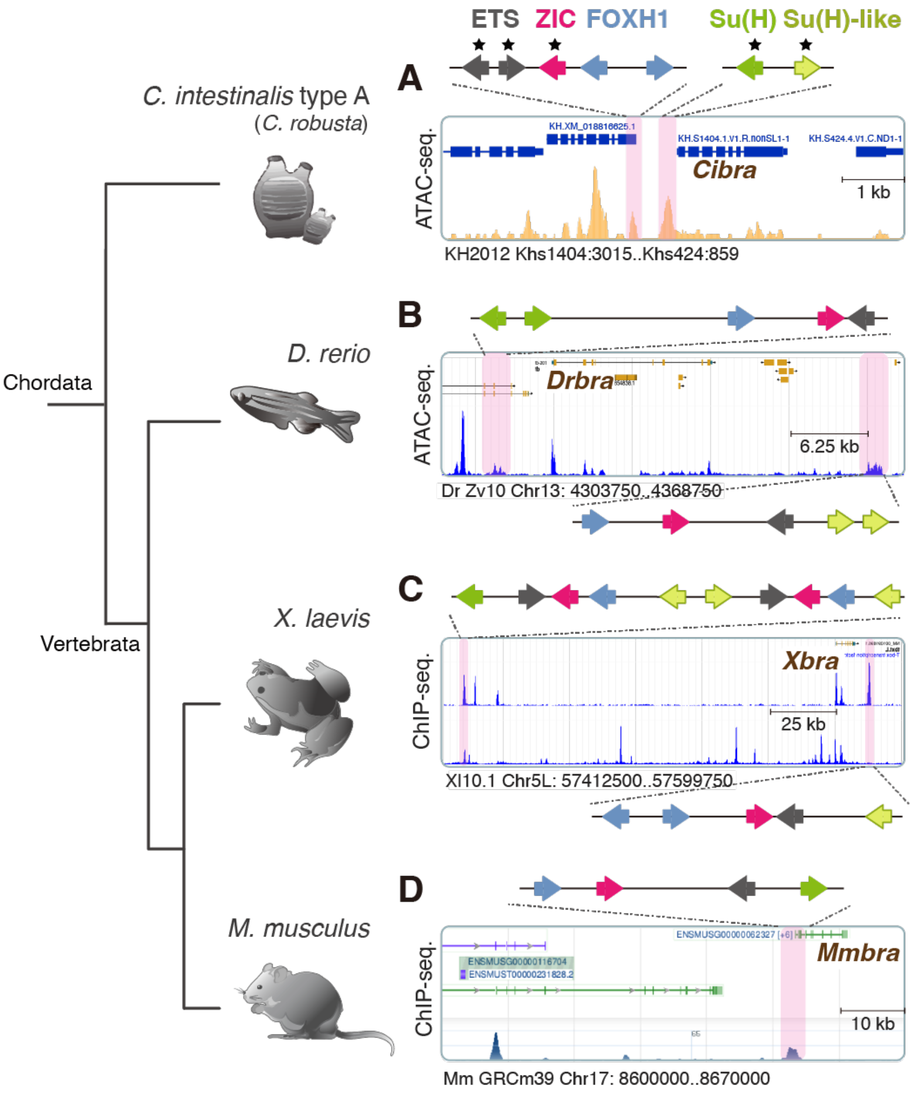

Fig S7. SFZE-containing CRMs in the *brachyury* loci of chordates. (**A-B**) The open chromatin regions were revealed by ATAC-seq at the *Cibra* and *Drbra* loci. Previously identified TF binding sites in the notochord enhancer of *Cibra* are indicated by the black asterisks. (**C-D**) Tracks display ChIP-seq of histone methylation (H3K4me3: upper track of C and the track in D) and Ets (lower track in C) at the *brachyury* loci of frog and mouse. CRMs highlighted by pink rectangles harboring the SFZE syntax. Orientations and orders of the TF binding sites are illustrated with colored arrows on top of the respective diagrams. The color key is indicated in panel A. Genome version, scaffold/chromosome number, and position of the genomic loci are displayed below the respective panel. Developmental stages or tissues/cells used for the ATAC-seq or ChIP-seq analyses are listed in table S7. Illustrations of *Xenopus* and mouse are adapted from BioRender. https://BioRender.com/14o3zg1

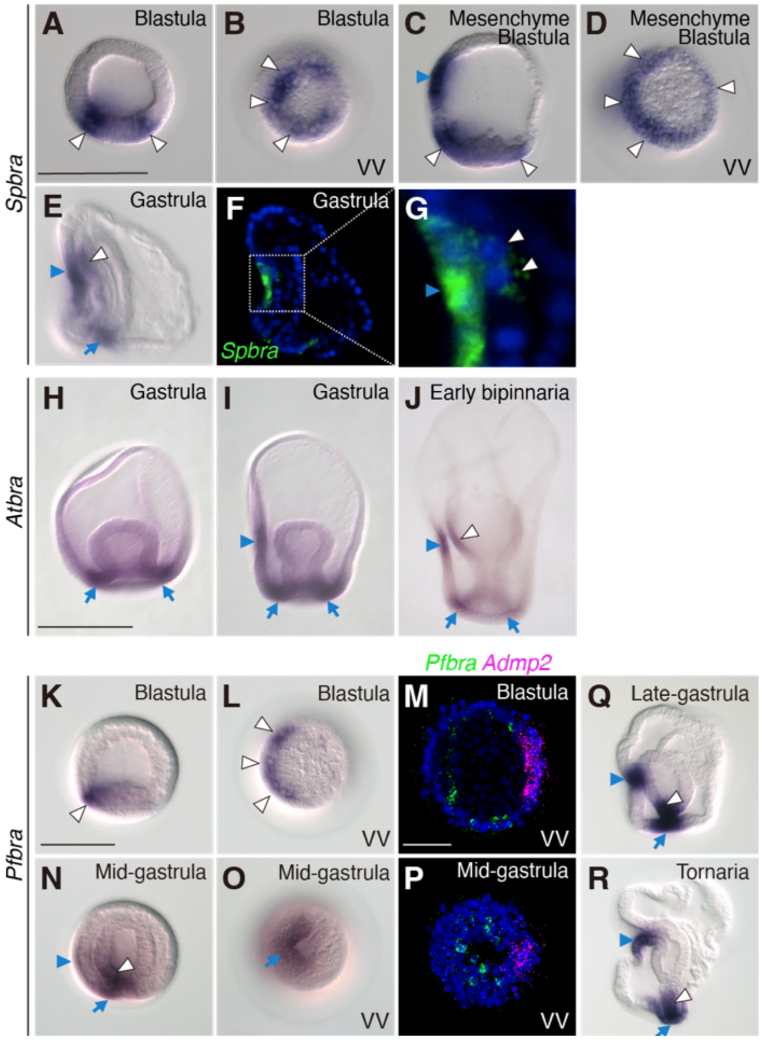

Fig S8. Expression of *brachyury* orthologs in ambulacrarians. (**A-G**) As previously described (16), during sea urchin embryogenesis, *Spbra* expression was initially detected in the vegetal region (white arrowhead) of the blastula (A-B), with an additional domain in the oral ectoderm (blue arrowhead) at the mesenchyme blastula stage (C-D). Panels B and D display the vegetal view of the same embryos in panels A and C, respectively, revealing enhanced *Spbra* expression on one side of the vegetal plate. The side with enhanced *Spbra* expression is the oral (ventral) side, judging from its known oral ectodermal expression. (**E**) At the late gastrula stage, *Spbra* was expressed in the oral ectoderm, cells surrounding the blastopore (blue arrow), and a few cells in the ventral archenteron (white arrowhead). (**F-G**) FISH of *Spbra* (green). The oral region of panel F is magnified in panel G, highlighting *Spbra* expression in the ventral archenteron (white arrowhead). (**H-J**) Expression of *Atbra* in the sea star *Archaster typicus*. The expression domain in the cells near the blastopore (blue arrow) and the oral ectoderm (blue arrowhead) during gastrulation. An additional expression domain in the ventral archenteron was detected at the early bipinnaria stage (white arrowhead). (**K-R**) *Pfbra* expression during hemichordate embryogenesis. *Pfbra* transcripts are detected on one side of the vegetal region (white arrowhead) at the blastula stage (K-L). By the mid-gastrula stage (N-O), *Pfbra* expression appears in the oral ectoderm (blue arrowhead), with stronger expression observed in the ventral

posterior archenteron (white arrowhead) and the ventral blastopore (blue arrow). Vegetal view of the panels K and N are displayed respectively in panels L and O. Double FISH of *Pfbra* (green) with a dorsal marker *Pfadmp2* (81) (magenta) confirms that the asymmetrical vegetal expression is on the ventral side (M and P). As previously described (17), *Pfbra* is expressed in the hindgut, oral ectoderm, and blastopore at the late gastrula and tornaria stages (Q-R). Nuclei were counterstained with Hoechst 33342 (blue) in panels F, G, M, and P. All scale bars represent 100 µm. Panels A-G, panels H-J, panels K-L, N-O and Q-R, and panels M-P are in the same scale, respectively. VV: vegetal view.

P. flava	GFP+ embryos (%)		Injected embryos
Pfbra:gfp BAC	5	25.0%	20
	2	11.8%	17
	4	57.1%	7
	5	45.5%	11
	1	11.1%	9

	Mesenchyme blastula							Late gastrula						
S. purpuratus	Presumptive ectoderm (%)		Presumptive endoderm (%)		Mesenchyme cells (%)		GFP+ embryos	Oral ectoderm & Blastopore (%)		Archenteron (%)		Mesenchyme cells (%)		GFP+ embryos
Spbra:gfp BAC	0	0.0%	48	100.0%	0	0.0%	48	36	85.7%	40	95.2%	6	14.3%	42
	4	5.9%	67	98.5%	0	0.0%	68	8	11.3%	71	100.0%	9	12.7%	71
	10	10.2%	93	94.9%	0	0.0%	98	37	60.7%	55	90.2%	5	8.2%	61

Table S1. Reporter activities of BACs in hemichordate and sea urchin embryos

	Shield			Early segmentation		
**Zebrafish**	Shield (%)		GFP$^+$ embryos	Dorsal midline (%)		GFP$^+$ embryos
Pfbra:gfp BAC	6	20.7%	29	6	46.2%	13
	6	66.7%	9	6	50.0%	12
	6	19.4%	31	3	37.5%	8
	6	24.0%	25			

	Shield			Early segmentation		
**Zebrafish**	Shield	GFP$^+$ embryos	Injected embryos	Dorsal midline	GFP$^+$ embryos	Injected embryos
Spbra:gfp BAC	0	0	48	Blurred expression		44
	0	0	66			50
	2	2	60			48

Table S2. Reporter activities of BACs in zebrafish embryos

	Shield						Early segmentation					
Zebrafish	Notochord progenitors (sum)		% (Overall average)		EGFP⁺ embryos (sum)		Notochord (sum)		% (Overall average)		EGFP⁺ embryos (sum)	
Pfbra promoter	13		41.9%		31		0		0.0%		20	
	20		46.5%		43		0		0.0%		25	
	18	(51)	46.2%	(45.1%)	39	(113)	0	(0)	0.0%	(0.0%)	19	(64)
PfCRM1	10		32.3%		31		6		15.4%		39	
	7		38.9%		18		3		12.0%		25	
	16		37.2%		43		2		6.9%		29	
	22	(55)	38.6%	(36.9%)	57	(149)	5	(16)	13.2%	(12.2%)	38	(131)
PfCRM2	38		90.5%		42		17		33.3%		51	
	12		92.3%		13		16		32.0%		50	
	35	(85)	68.6%	(80.2%)	51	(106)	6		31.6%		19	
							10		33.3%		30	
							13	(62)	34.2%	(33.0%)	38	(188)
PfCRM3	3		8.3%		36		0		0.0%		34	
	2		4.5%		44		0		0.0%		22	
	3		7.3%		41		0	(0)	0.0%	(0.0%)	27	(83)
	1	(9)	3.1%	(5.9%)	32	(153)						
PfCRM4	6		15.4%		39		0		0.0%		41	
	14		31.1%		45		1		1.8%		56	
	8	(28)	33.3%	(25.9%)	24	(108)	0	(1)	0.0%	(0.8%)	33	(130)
Drntl-1 kb	36		94.7%		38		12		42.9%		28	
	41		100.0%		41		5		35.7%		14	
	57	(134)	98.3%	(97.8%)	58	(137)	16	(33)	64.0%	(49.3%)	25	(67)

Table S3. Reporter activities of PfCRMs in zebrafish embryos

Species	CRMs	Span of FZE (bp)	Span of SFZE (bp)
C. owczarzaki	CoCRM4	101	218
N. vectensis	NvCRM1	160	741
	NvCRM2	175	406
	NvCRM3	116 / 180	229 / 434
Drosophila	DmCRM1	122	911
S. purpuratus	SpCRM4	124	478
P. flava	PfCRM2	139	299
B. floridae	*Bfbra1* SFZE	57	396
C. robusta	*Cibra* SFZE	33	525
D. rerio	*Dmtl* - 1kb	56	99
	Drbra 5'SFZE	118	864
	Drbra 3'SFZE	89	116
X. laevis	*Xbra* 5' SFZE	105 / 143	270 / 436
	Xbra 3' SFZE	95	229
M. musculus	*Mmbra* SFZE	127	170

Table S4. Spans of FZE and SFZE in various organisms

Zebrafish	Early segmentation					
	Notochord (sum)		% (Overall average)		EGFP$^+$ embryos (sum)	
SpCRM4	3		8.6%		35	
	8		16.7%		48	
	5	(16)	16.1%	(14.0%)	31	(114)
DmCRM1	10		37.0%		27	
	13		68.4%		19	
	11		52.4%		21	
	4	(38)	44.4%	(50.0%)	9	(76)
NvCRM1	8		21.1%		38	
	4		14.8%		27	
	5	(17)	21.7%	(19.3%)	23	(88)
NvCRM2	5		17.2%		29	
	9		18.0%		50	
	8	(22)	22.9%	(19.3%)	35	(114)
NvCRM3	19		36.5%		52	
	16		30.8%		52	
	22	(57)	34.9%	(34.1%)	63	(167)
CoCRM4	12		22.2%		54	
	6		18.8%		32	
	10	(28)	16.4%	(19.0%)	61	(147)

Table S5. Notochord activities of SFZE-containing CRMs in zebrafish embryos

Zebrafish	Early segmentation: Notochord (sum)		% (Overall average)		EGFP$^+$ embryos	
PfCRM2 w/oFOXH1	10		25.0%		40	
	17		29.3%		58	
	11		22.0%		50	
	11	(49)	22.4%	(24.9%)	49	(197)
PfCRM2 w/oFOXH1-V	5		17.9%		28	
	7		18.9%		37	
	5	(17)	18.5%	(18.5%)	27	(92)
PfCRM2ΔETS	4		11.4%		35	
	1		2.2%		46	
	1	(6)	2.1%	(4.7%)	47	(128)

S. purpuratus	Mesenchyme blastula: Ectoderm (%)		Presumptive endoderm (%)		Mesoderm (%)		EGFP$^+$ embryo	Injected embryos	Late gastrula: Non-oral ectoderm		Archenteron (%)		Mesenchyme cells (%)		EGFP$^+$ embryos	Injected embryos
Spbra promoter	0		0		0		0	66	4		0		3		6	67
	5		2		0		7	61	0		0		0		0	68
	3		0		0		3	66	4		0		3		7	59
SpCRM4	16	23.2%	62	89.9%	6	8.7%	69		12	33.3%	23	63.9%	8	22.2%	36	
	10	9.8%	100	98.0%	0	0.0%	102		15	46.9%	24	75.0%	2	6.3%	32	
	12	21.1%	54	94.7%	0	0.0%	57		10	35.7%	19	67.9%	6	21.4%	28	
	7	15.6%	43	95.6%	0	0.0%	45		17	29.3%	49	84.5%	13	22.4%	58	
SpCRM4 ΔFOXH1	2	3.6%	55	100.0%	2	3.6%	55		20	50.0%	16	40.0%	17	42.5%	40	
	6	20.7%	28	96.6%	1	3.4%	29		6	31.6%	7	36.8%	7	36.8%	19	
	3	11.1%	25	92.6%	1	3.7%	27		9	29.0%	17	54.8%	15	48.4%	31	
SpCRM4 ΔETS	12	30.0%	31	77.5%	1	2.5%	40		20	46.5%	29	67.4%	7	16.3%	43	
	10	22.2%	30	66.7%	0	0.0%	45		15	31.9%	38	80.9%	14	29.8%	47	
	7	21.2%	26	78.8%	1	3.0%	33		25	58.1%	23	53.5%	14	32.6%	43	

Table S6. Activities of reporters without Foxh1 or Ets binding sites in zebrafish and sea urchin embryos

Organisms	Analytic type	Developmental stages/ Tissues/ Cells	Reference
C. owczarzaki	ChIP-seq	Filopodia	54
C. robusta	ATAC-seq	Late gastrula	62-64
D. rerio	ATAC-seq	80% epiboly	52
X. laevis	ChIP-seq	NF 10.5 and NF 11.5	61
M. musculus	ChIP-seq	Primary tissues, primary cells and immortalized cell lines	60
P. flava	ATAC-seq	Early blastula	55
S. purpuratus	ATAC-seq	Late gastrula	65
Drosophila	ChIP-seq	Embryo 6-8 h	66
N. vectensis	ChIP-seq	Gastrula	53

Table S7. ATAC-seq and ChIP-seq datasets used in this study

CRMs / Genes	Direction	Sequences
GFP knock-in BAC	F	CAACACATCGTGACTTGTTCCTTCTCTGTCTCCAGACCTAGCATGAGCAAGGGCGAGGAACT
	R	ATTTCAGCGTGCATTTTGTTTTTTTTAGCGTGTCGTGTATCTCCTCGAAGAGCTATTCCAGAAGTAGTGA
Pfbra promoter	F	GAATTCTGTGCAGCCAATAGGAGCG
	R	TGCTTACCATGGCTAGGTCTGGAGACAGAGAAG
PfCRM1	F	TAATGAGGGCCCGCAGAAAATCAACACGTG
	R	TGCACAGAATTCGTTCATAAAGCCGATCATG
PfCRM2	F	TAATGAGGGCCCTTCAGTTCCAGCCCGAGTTAC
	R	TGCTTACCATGGCTAGGTCTGGAGACAGAGAAG
PfCRM3	F	GGGCCCTGACTGGCCACTTGGCAG
	R	TGCACAGAATTCGAAAGTGTAGCAAAGACGATCTCC
PfCRM4	F	GGGCCCACATCGGGAAAGCTATGAGTAG
	R	TGCACAGAATTCCTAAACTGAAGGTGGCGTCA
Spbra promoter	F	GAATTCGAACAGTTGACCAATCACGC
	R	CCATGGAGTGGCGTTGATGGGTTG
SpCRM4	F	TAATGAGGGCCCTGTCGCCTCTCTCTTTTTCAAAC
	R	GAATTCCATGTGTGATTCTTGCGTAATCC
DmCRM1	F	TAGTTTAAGGGTTTTTGTTAGTGAATATCTTG
	R	TGAAACCAAAATTGAGATAAAGTGC
NvCRM1	F	CCCTTGTCTGCGTTCAATATC
	R	GTGAGAGGACGAGTGGC
NvCRM2	F	TTGCGGAATTGATATTATTAGAACTATGG
	R	TATCAAAATAATTCATAACCTCGGAAACA
NvCRM3	F	TCGGTGAAATTGCTGTTTGAAAT
	R	CCGACTATGCTTGTTCTTTTGC
PfCRM2w/oFOXH1	F	CAAACTACCCAGCCCTACCGTT
	R	TGCTTACCATGGCTAGGTCTGGAGACAGAGAAG
PfCRM2w/oFOXH1-V	F	TAACAGGGTAATGAGGGCC
	R	ACCTCTTCCCGCATCG
PfCRM2ΔETS	F	CAAATATTGCTAAAATTCCCACGTCAAG
	R	TCATGTACATAATTGCACATGGGC
SpCRM4ΔFOXH1	F1	TTATGGACCAAAAATAGAGGCTTTGC
	R1	GTCCGCTTAACTGCGCG
	F2	CCCTTTTAAGAATCTGTCTTGAAACAATTT
	R2	TCATCCAGAACTACAACAATCTACGAC
SpCRM4ΔETS	F	TCGCGTCAATAATCAATCGATCGC
	R	TTAAAGTTGCGCTCAACACCAGAC
gfp	F	GAGCAAGGGCGAGGAACT
	R	CCCAGCAGCGGTCACAAAC
egfp	F	CAAGGGCGAGGAGCTGTTC
	R	GGTGGCGGCCGCTTTAC
Drntl	F	CTCGGTGGCTGGTTCCTG
	R	GATTGTCTCTCATCTTGATTGTAAATACG
Drmnx1	F	TACGAAGAGGAGCGGTGACA
	R	CCCTTTTGCTTCTCGGCATC
Pfbra	F	TCAATTGGGTGGATGGTTTTTG
	R	CAGCCTCGTTCTCGCTATTC
Spadmp2	F	TGAACACCTGCCACCTGAAGTTACAC
	R	GTTCAGGCGTGAATAGAAACCGAGTC
Atbra	F	TGTCCGCGGACACCATGCG
	R	TCAAACTCCGGGAGAAGGTGGTG

Table S8. Primers used to generate reporter constructs and *in situ* hybridization probes
